# Optimization-driven machine learning intrusion detection systems for resource-constrained internet of things challenges: a systematic review and future directions

**DOI:** 10.7717/peerj-cs.4048

**Published:** 2026-10-27

**Authors:** Feras Mohammed Al-Matarneh, Bassam Abdo Al-Hameli

**Affiliations:** 1 Faculty of Information Technology, Department of Computer Science, Isra University, Amman, Jordan; 2 Information Technology Department, AL-Madinah International University, Taman Desa Petaling, Kuala Lumpur, Malaysia; 3 Department of Information Systems, Islamic University of Minnesota, Minneapolis, MN, United States of America

**Keywords:** Internet of things (IoT) attacks, Intrusion detection system (IDS), Machine learning (ML), Deep learning

## Abstract

The explosive growth of the Internet of Things (IoT) across smart homes, healthcare, industrial systems, and critical infrastructure has dramatically enlarged the cyberattack surface, rendering traditional security mechanisms ineffective against evolving threats in highly heterogeneous and resource-constrained environments. This article presents a comprehensive and systematic review of machine learning (ML) and deep learning (DL) based intrusion detection systems (IDSs) tailored for IoT, with a particular focus on optimization algorithms for feature selection (FS), hyperparameter tuning, and model lightweighting. We critically analyze state-of-the-art methodologies, benchmark datasets, deployment strategies, and performance trade-offs, while uncovering previously hidden correlations between optimization techniques and ML/DL models that significantly enhance detection accuracy, interpretability, energy efficiency, and real-time feasibility on edge devices. The effectiveness of these approaches is systematically evaluated across diverse IoT applications, including real-time anomaly detection, lightweight edge deployment, and encrypted traffic analysis. Key challenges in integrating advanced ML/DL into constrained IoT environments are thoroughly examined, and a forward-looking research framework is proposed to overcome current limitations and drive the development of the next generation of intelligent, adaptive, and resilient IoT-IDS solutions.

## Introduction

The Internet of Things (IoT) describes a vast and dynamic network of interconnected physical devices from sensors and actuators to smart appliances and industrial controllers that gather, exchange, and act on data *via* the internet. The most accurate and current Statista projection aligns with approximately 19.8 billion IoT connections worldwide in 2025, expected to grow to over 40 billion by 2034 (Younus et al., 2025), IoT has become a cornerstone of modern smart homes, healthcare systems, agricultural technology, and Industry 4.0 innovations, see Fig. 1. Despite its widespread adoption, the decentralized and heterogeneous nature of IoT ecosystems, combined with resource constraints of many edge devices, creates significant security vulnerabilities. Traditional protections like firewalls and encryption often fall short in these environments (Cui et al., 2024; Li et al., 2025). As a result, Intrusion Detection Systems (IDS) have become essential for monitoring network and device behaviors, enabling real-time detection of malicious activities to prevent data loss and physical damage. In specific sectors such as maritime shipping communications, IoT’s deep integration requires highly stable and robust architectures (Farah et al., 2024). Conventional machine learning-based IDS approaches relying on centralized training present challenges, including risks of data privacy breaches and communication overhead, particularly in remote maritime settings. These limitations are driving efforts to develop decentralized IDS frameworks better suited to the unique constraints of IoT networks in complex environments (He et al., 2024).

**Figure 1:**
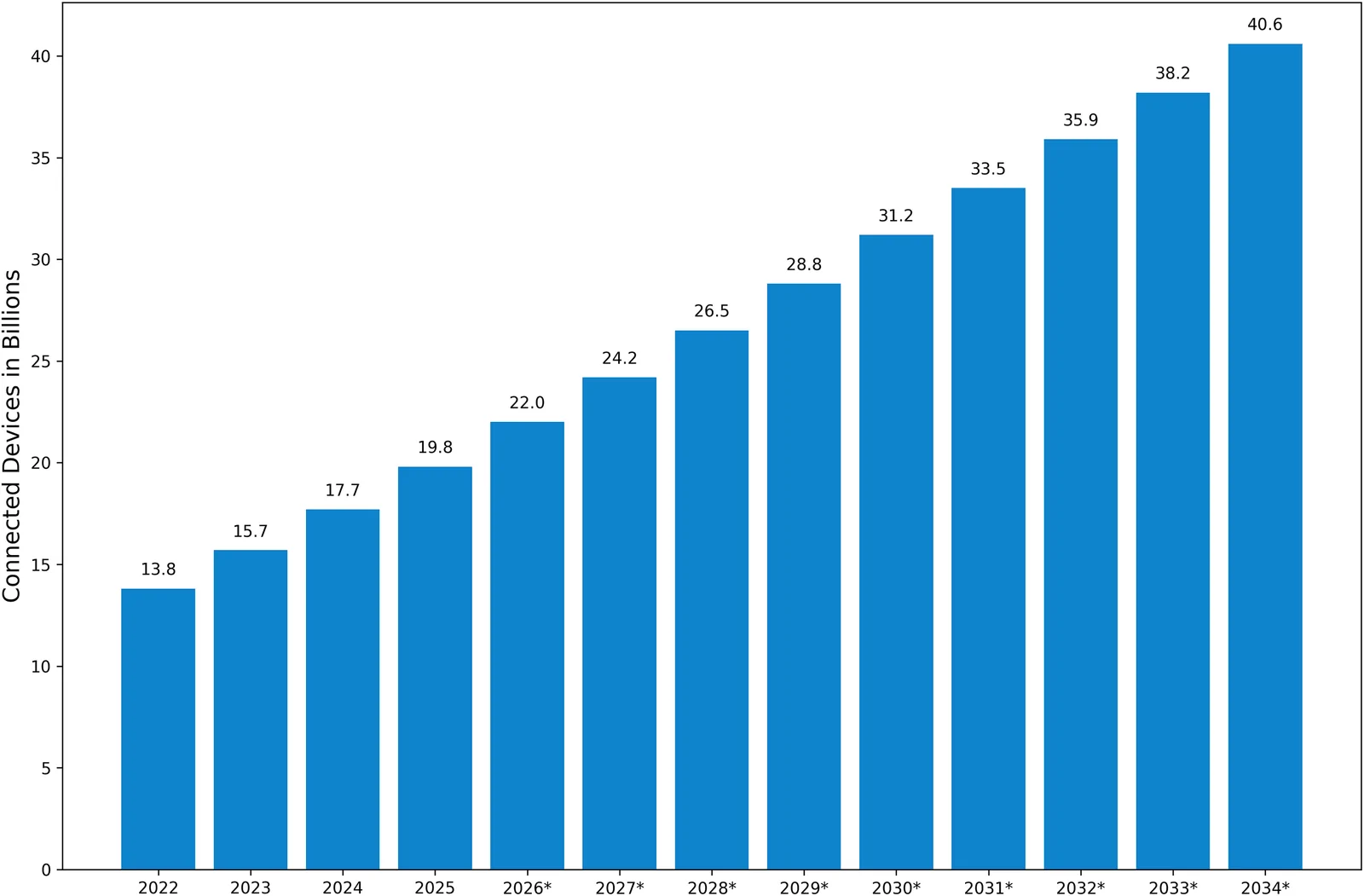
Number of IoT connections worldwide from 2022 to 2034. *Projected data. Source: statista.com.

A substantial share of the most prevalent IoT threats continues to consist of various Mirai DDoS botnet variants, as illustrated in Fig. 2. During the first quarter of 2025, BitCoinMiner maintained active distribution, accounting for 7.32% of detected threats. Meanwhile, attacks involving the NyaDrop botnet decreased, comprising 19.31% of detections compared to the fourth quarter of 2024 (AMR, 2025).

**Figure 2:**
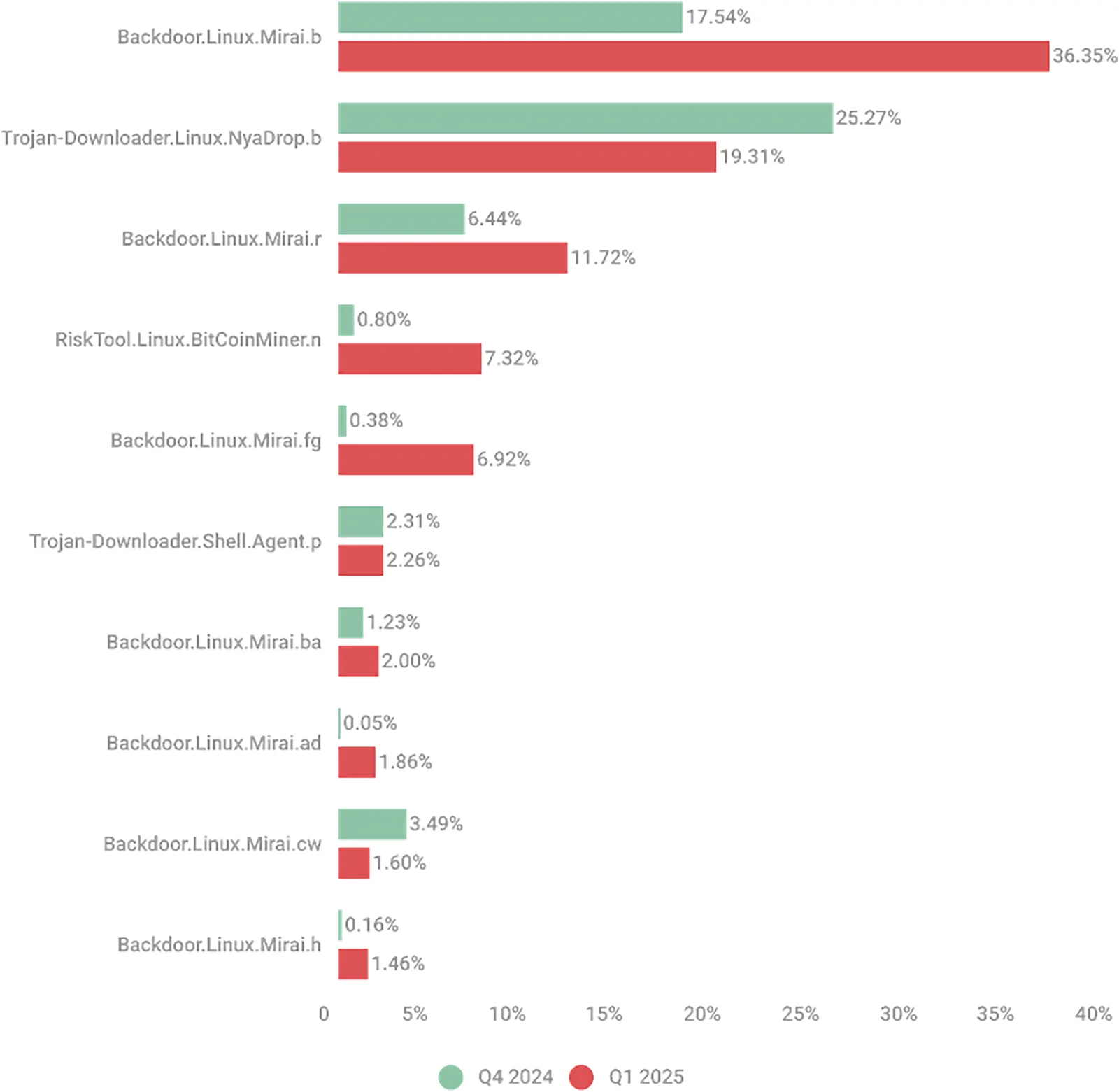
Top 10 threats delivered to IoT devices. Source: Securelist, malware report Q1 2025.

The statistics presents an analysis of the geographical distribution of sources behind online attacks targeting IoT networks. To identify the origin of these attacks, domain names were resolved to their actual IP addresses, and the geographical location of those IPs was determined using GeoIP technology. In the first quarter of 2025, security solutions blocked over 629 million attacks originating from internet resources worldwide. Additionally, web anti-virus tools detected approximately 88 million unique URLs involved in malicious activities, as shown in Fig. 3. This data underscores the vast and diverse threat landscape facing IoT networks globally, highlighting the critical need for robust intrusion detection systems capable of addressing geographically dispersed cyber threats (AMR, 2025).

**Figure 3:**
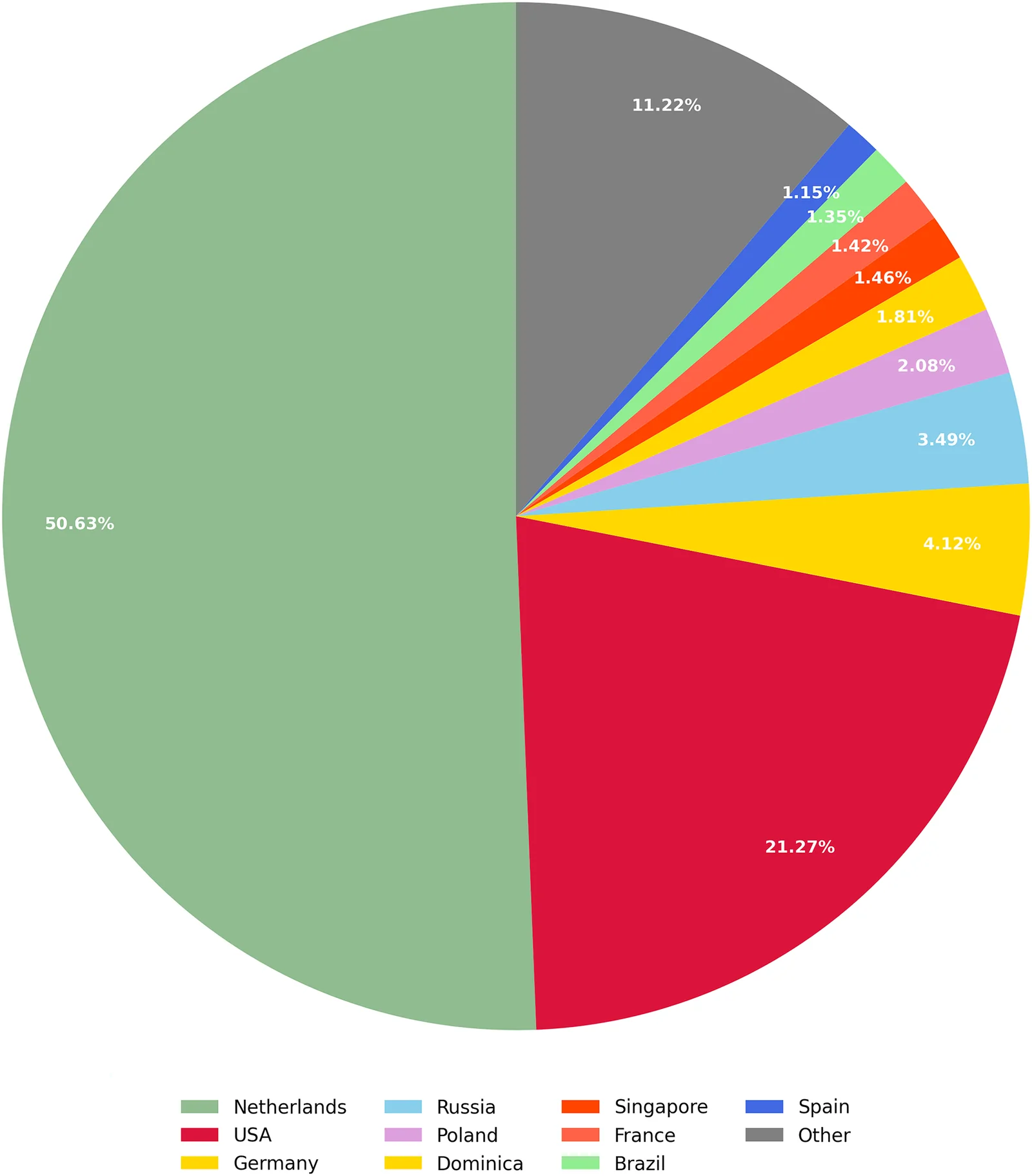
Distribution of web-based attack sources by country, Q1 2025. Source: Securelist, malware report Q1 2025.

Given the rapid expansion of IoT ecosystems and the increasing sophistication of cyber threats, there is a pressing need for a consolidated and technically rigorous synthesis of recent advances in intrusion detection systems tailored for resource-constrained environments. While machine learning and deep learning techniques have shown strong detection capabilities, their practical deployment in IoT networks remains constrained by computational overhead, energy limitations, and real-time processing requirements. Existing surveys often address IDS architectures or learning models in isolation, without systematically examining how optimization techniques enable lightweight, efficient, and deployable solutions. This review addresses this gap by providing a comprehensive analysis of optimization-driven machine learning/deep learning (ML/DL)-based IDS frameworks. Accordingly, this work is particularly relevant for cybersecurity researchers, IoT system architects, machine learning practitioners, graduate students, and industry engineers seeking scalable, energy-efficient, and real-world deployable security solutions.

This study focuses on the most recent and impactful techniques in IoT intrusion detection, specifically targeting peer-reviewed research published between 2015 and 2025. By concentrating on this time frame, the review distinguishes itself from earlier surveys and ensures coverage of state-of-the-art developments. Irrelevant and duplicate studies were systematically excluded to maintain quality and rigor. Furthermore, research involving metaheuristics and ML-driven detection systems integrated with blockchain, fog computing, or supercomputing technologies was excluded to preserve a clear focus on resource-constrained IoT environments. Ultimately, 118 high-quality articles were selected and analyzed to provide a comprehensive overview of current advancements, emerging trends, and future research directions in IoT intrusion detection systems.

## Survey methodology

This review follows a systematic and transparent approach to analyze recent research on IoT intrusion detection systems (IDS). Peer-reviewed articles published between 2018 and 2025 were retrieved from IEEE Xplore, Scopus, and Web of Science (WoS) using focused search queries targeting machine learning and deep learning-based IDS. Duplicates and irrelevant articles were removed, and the remaining studies were screened based on predefined inclusion and exclusion criteria. Specifically, studies were included if they addressed IoT environments, proposed or evaluated IDS techniques, and utilized publicly available or well-documented datasets, while works lacking methodological clarity or not directly related to IoT-IDS were excluded. The selected studies were then systematically analyzed in terms of IDS models, detection techniques, deployment strategies, performance metrics, and security challenges. For dataset filtering, all datasets reported in the selected studies were collected and organized into a structured taxonomy based on characteristics such as data source, traffic type, attack diversity, and IoT relevance. Furthermore, the review critically evaluates the suitability of these datasets for resource-constrained IoT environments, highlights the limitations of obsolete benchmarks such as NSL-KDD and early CIC datasets that lack IoT protocol specificity, and introduces a Trade-off Analysis to guide researchers in selecting appropriate datasets according to their research focus (*e.g*., edge-level *vs*. cloud-level IDS). This methodology ensures a comprehensive, unbiased, and reproducible overview of current advances in IoT intrusion detection.

### Methodological framework for the systematic review

The objectives of this systematic literature review are defined as follows: first, to identify and analyze optimization-driven machine learning and deep learning approaches applied to intrusion detection systems in resource-constrained IoT environments; second, to examine the optimization strategies employed, including feature selection, hyperparameter tuning, and model lightweighting techniques; and third, to evaluate the performance metrics and deployment considerations used to assess the effectiveness and feasibility of these approaches. Unlike previous surveys that focus on attack types, the study provides a rigorous mapping of optimization algorithms to specific ML/DL architectures to solve the resource-latency trade-off.

Although numerous studies have proposed ML/DL-based intrusion detection systems for IoT networks, only a limited number of reviews systematically focus on the role of optimization techniques in enabling lightweight and deployable solutions. Moreover, few works comprehensively analyze evaluation metrics alongside computational cost, scalability, and energy consumption, which are critical factors in resource-constrained environments. This lack of consolidated analysis highlights the need for a structured and targeted review. A formal review protocol was established to guide the systematic process and ensure transparency and reproducibility. Research articles were retrieved using two predefined query strings applied to IEEE Xplore, WOS Digital Library, and Scopus. The retrieved studies were then screened and reviewed through a multi-stage filtering process. Inclusion and exclusion criteria were applied to ensure relevance and quality. Subsequently, the selected articles were tabulated, and the contributions of each study were extracted and documented. The articles were classified based on IDS architecture, learning models, and optimization techniques. Performance metrics, computational overhead, and deployment environments were analyzed, and quality attributes such as accuracy, efficiency, scalability, and real-time feasibility were specified. The detailed components of the review protocol are presented in the following sections.

1. Databases: To ensure a comprehensive, transparent, and unbiased review of the literature, a systematic search strategy was employed across multiple reputable digital libraries and indexing databases, including IEEE Xplore, Scopus, Web of Science, ScienceDirect, ACM Digital Library, SpringerLink, and Google Scholar. These sources were selected to maximize coverage of high-impact journals and conference proceedings in the fields of cybersecurity, IoT, and machine learning. The search was conducted using carefully designed keyword combinations and Boolean operators related to IoT intrusion detection systems, machine learning and deep learning models, and optimization techniques. This approach ensured the retrieval of a broad and relevant set of publications addressing both theoretical and practical aspects of IoT security.
2. Search strategy: Two query strings were designed to systematically retrieve literature on optimization-driven ML-based intrusion detection for IoT. Query String 1 (QS1) combined terms for IoT, IDS, machine/deep learning, and optimization techniques (*e.g*., feature selection, hyperparameter tuning, model compression, lightweight design) to capture a broad range of relevant studies. Query String 2 (QS2) refined the focus by adding “method,” “technique,” or “methodology” to identify articles proposing explicit optimization frameworks. Both queries used standardized Boolean logic and were applied to IEEE Xplore, ACM Digital Library, and Scopus, searching titles, abstracts, and keywords. Retrieved studies were analyzed for optimization strategies, ML models, evaluation metrics, and deployment feasibility in resource-constrained IoT settings.
3. Quality assessment: To ensure the quality and relevance of selected studies, strict inclusion and exclusion criteria were applied. Only peer-reviewed journal articles and conference articles published between 2015 and 2025 were considered. Studies were required to propose or evaluate machine learning or deep learning-based intrusion detection systems specifically designed for IoT environments and to incorporate optimization techniques aimed at improving performance or reducing computational overhead. Articles that were not peer-reviewed, lacked experimental validation, focused solely on traditional IDS, or integrated blockchain, fog computing, or supercomputing architectures were excluded to maintain the scope of this review.
4. Screening process: A three-stage screening process was conducted to filter the retrieved publications. First, titles were examined to remove clearly irrelevant works. Second, abstracts were reviewed to assess relevance to the research objectives. Finally, full-text assessments were performed to ensure methodological rigor and alignment with the inclusion criteria. This systematic filtering process resulted in the final selection of 118 high-quality studies for detailed analysis.
5. Data extraction: For each selected article, a structured data extraction process was applied to capture essential information, including IDS architecture, machine learning model type, optimization technique employed, dataset used, feature engineering strategy, evaluation metrics, computational overhead, and deployment environment. The extracted data were then synthesized qualitatively and quantitatively to identify performance trends, compare optimization strategies, classify IDS architectures, and highlight research gaps. This systematic methodology ensures the reliability, reproducibility, and scientific rigor of the review findings.
6. Synthesis method: A combined qualitative and quantitative synthesis was conducted across the 118 studies to identify performance trends, compare optimization strategies (*e.g*., neural networks (NN), support vector machines (SVM), and random forests (RF)), and classify IDS architectures (centralized, edge-based, hybrid). This analysis revealed key gaps such as overreliance on outdated datasets, limited real-world validation, and insufficient focus on model interpretability and informed concrete recommendations for future research toward lightweight, robust, and deployable IoT intrusion detection systems.

## Threat landscape in IoT networks

The rapid growth of the Internet of Things is transforming a variety of important application domains, each defined by factors such as network reach, coverage, scale, device diversity, how often tasks recur, user engagement, and overall impact. The structured taxonomy demonstrates how IoT transcends traditional boundaries, creating interconnected ecosystems that deliver measurable value across environmental sustainability, public health, energy efficiency, industrial productivity, and consumer convenience. The convergence of specialized sensing networks (wireless sensor networks (WSNs), body area networks (BANs)), domain-specific platforms (Smart Home, industrial Internet of Things (IIoT), and Internet of Health Things (IoHT)), and scalable cloud/edge infrastructures forms the backbone of the intelligent, data-driven world of tomorrow (Castro, Deng & Park, 2023; da Silva, de Souza & Travassos, 2024). The Fig. 4 presents a structured overview of IoT applications, grouping them into five main domains including Smart Living, Smart Healthcare, Smart Industry, Smart Cities, and Smart Agriculture, each with representative use cases. While these categories help organize the landscape, the boundaries between them are often fluid, as many IoT applications and their data span multiple domains, highlighting the interconnectedness and versatility of IoT systems in both daily life and industry.

**Figure 4:**
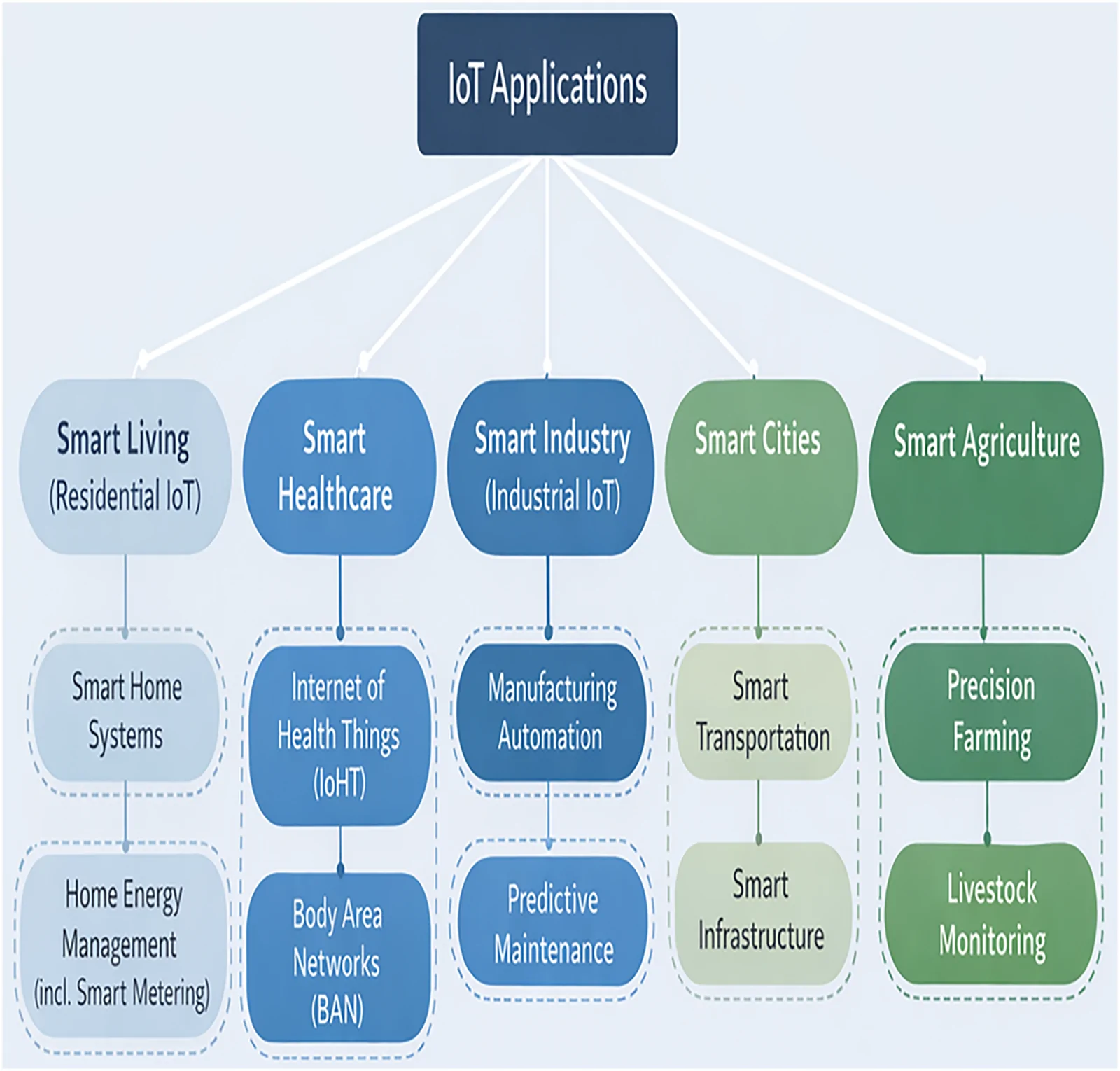
IoT applications and their domains.

The rapid expansion and inherent heterogeneity of the Internet of Things have significantly broadened its exposure to security risks. Unlike traditional IT environments supported by mature and widely adopted security standards, the IoT domain continues to lack unified security frameworks, leaving it particularly susceptible to a wide range of adversarial actions (Taherdoost, 2023). The study presents a comprehensive synthesis of the threat landscape in IoT networks, derived from a systematic review of many peer-reviewed publications for 45 attacks types have classified (see Table 1). It offers a structured taxonomy of prevalent cyber threats targeting IoT ecosystems, categorizing each by attack type, description, impact, common targets, and supporting literature.

**Table 1:** IoT attack taxonomy.

Attack type	Attack description|impact type|common targets	Scheme
Botnet (BN)	Compromising devices in large numbers for coordinated attacks|service disruption, DDoS|Routers, cameras, home devices	Suganth & Usha (2018)
Brute force (BF)	Attempting password combinations to gain unauthorized access|unauthorized access|smart home devices	Bonandrini, Bercher & Zangar (2019)
Buffer reservation	Exploiting buffer allocation weaknesses|device crash, data corruption|embedded systems	Krishna et al. (2021)
Deauthentication	Forcing devices to lose network association|service interruption|smart home devices, Routers	Alam & Tomai (2023)
Delay attack	Intentionally delaying packet delivery to disrupt services|service degradation|sensor networks, IoT Routers	Deogirikar & Vidhate (2017)
Denial of service (DoS)	Overloading a device or service to make it unavailable|service outage|all IoT sectors	Mohamed & Aydin (2022)
Distributed DoS (DDoS)	Large-scale coordinated overload of IoT targets|availability loss|cameras, Routers, industrial IoT	Suganth & Usha (2018)
Eavesdropping	Listening to network traffic to steal sensitive data|data leakage, privacy loss|healthcare, smart home devices	Msgna (2022)
Man-in-the-middle	Intercepting or modifying communication between devices|data modification, theft|smart home devices, healthcare	Elrawy, Awad & Hamed (2018)
Replay attack (RP)	Reusing captured packets to gain access|unauthorized access, data theft|sensors, home automation	Alam & Tomai (2023)
Ransomware	Encrypting data and demanding ransom|service unavailability, data loss|healthcare, smart homes	Msgna (2022)
Spoofing (SP)	Forging identity to gain device or network access|unauthorized access, data theft|smart meters, industrial IoT	Bonandrini, Bercher & Zangar (2019)
SQL injection	Injecting malicious SQL queries *via* applications|data Loss, System Compromise|IoT Gateways, Web APIs	Alam & Tomai (2023)
Tampering	Unauthorized modification of hardware or software|device sabotage, data loss|industrial devices, smart meters	Kermani et al. (2013)
User-to-root (U2R)	Escalating user privileges to admin level|full control, data manipulation|all IoT devices	Msgna (2022)

The analysis reveals that IoT threats span the entire attack surface from physical-layer manipulations (*e.g*., Hardware Trojan, Physical Sensor Tampering) to network-layer disruptions (*e.g*., Jamming, Sinkhole, Flooding) and application-layer exploits (*e.g*., SQL Injection, XSS, ransomware). A significant portion of attacks focus on credential abuse and access control bypass, including Brute Force, SSH/FTP Patator, Masquerade, and Privilege Escalation, highlighting the persistent weakness in authentication mechanisms across IoT devices (Shah et al., 2023; Sharafaldin, Lashkari & Ghorbani, 2018). Moreover, the Table 1 underscores the diverse impact profiles of these threats: beyond traditional confidentiality breaches, many attacks induce service unavailability (*e.g*., DoS, DDoS, sleep deprivation), data integrity violations (*e.g*., Replay, Elevation of Privilege) (Makhdoom, 2020), or physical-layer consequences such as device malfunction or energy exhaustion. Critically, the targets of these attacks reflect the heterogeneity of the IoT ecosystem from consumer devices like smart cameras and home assistants to industrial sensors, medical equipment, and critical infrastructure gateways. This universality of exposure emphasizes that no IoT domain remains immune, whether smart homes, healthcare (IoMT), industrial systems (IIoT), or urban infrastructure (Serkov et al., 2020; Umair et al., 2021).

This analysis reveals that the threat landscape in IoT is shaped by the ecosystem’s unique structural and operational traits. Prior work has introduced structured models to categorize attack surfaces according to network typologies and architectural layers, offering a multi-dimensional view of potential compromise points. Other studies have identified core attributes such as resource limitations, device diversity, operational autonomy, and pervasive deployment that collectively heighten susceptibility to malicious activity. Furthermore, certain device categories, due to their prevalence across multiple application domains, represent high-value targets and serve as critical focal points for understanding cross-sectoral threat patterns. Overall, this taxonomy not only maps the evolution and sophistication of IoT-specific threats over the past decade but also serves as a foundational reference for designing layered, context-aware defense strategies that account for the unique constraints and risks inherent in IoT environments.

## Intrusion detection systems and techniques

### Intrusion detection systems types

IDS is a critical cybersecurity mechanism designed to monitor, detect, and respond to unauthorized activities or policy violations within information systems. According to the National Institute of Standards and Technology (NIST), an IDS continuously analyzes system events and network traffic to identify suspicious patterns that may indicate a security breach or malicious intent (Szymanski, 2022). Unlike firewalls that primarily enforce access control policies, IDS focuses on detecting attacks that have bypassed perimeter defenses, thus serving as a second line of defense in depth-in-defense architectures (Drewek-Ossowicka, Pietrołaj & Rumiński, 2021; Kikissagbe & Adda, 2024). IDS can be broadly classified based on the source of data being monitored into three primary categories: Network-based Intrusion Detection Systems (NIDS) (Chua & Salam, 2022), Host-based Intrusion Detection Systems (HIDS) (Satımış, Akleylek & Tok, 2024), and Hybrid or Distributed IDS. More recently, Protocol-based Intrusion Detection Systems (PIDS) (de Souza et al., 2022; Li et al., 2021) and application-layer variants have emerged, particularly in IoT and cloud environments.

1. Network-based Intrusion Detection System (NIDS)
  A Network Intrusion Detection System (NIDS) monitors network traffic in real time by analyzing packets traversing network segments, typically at gateways, routers, or switches (Santos et al., 2023). NIDS operates in promiscuous mode, capturing and inspecting packet headers and payloads to detect known attack signatures or anomalous traffic patterns (Shobana & Geetha, 2025). Signature-based NIDS (*e.g*., Snort, Suricata) match traffic against a database of known attack patterns, while anomaly-based NIDS establish a baseline of normal behavior and flag deviations (Luay et al., 2025). High visibility across the network, centralized management, and ability to detect network-wide threats such as DDoS, port scans, and worm propagation. Inability to inspect encrypted traffic, high false positives in anomaly mode, and performance degradation under heavy loads (Ch & Yamarthi, 2025; Sreelakshmi et al., 2024).
2. Host-based Intrusion Detection System (HIDS)
  A Host-based Intrusion Detection System (HIDS) resides on individual hosts (servers, endpoints, or IoT devices) and monitors internal system activities, including system calls, file system modifications, log files, and application behavior (Zhang et al., 2022). Popular HIDS tools include OSSEC, Tripwire, and AIDE (Taha & Roy, 2024). HIDS excels at detecting insider threats, privilege escalation, rootkits, and file integrity violations that are invisible to network sensors (Celdrán et al., 2023). Deep visibility into endpoint behavior, effective against encrypted traffic attacks, and resilience to network spoofing. High resource consumption, lack of network-wide context, and vulnerability if the host is compromised (Zhaikhan et al., 2020).
3. Protocol-based Intrusion Detection System (PIDS)
  A Protocol-based Intrusion Detection System (PIDS) focuses on analyzing protocol-specific behavior at the application or transport layer, particularly in constrained environments such as IoT. PIDS enforces expected protocol state transitions (*e.g*., MQTT, CoAP, RPL) (Roihan, Priyambodo & Ashari, 2019) and detects violations such as malformed packets, unauthorized PUBLISH commands, or incorrect DODAG versioning in 6LoWPAN networks (Cheng et al., 2025). In IoT, PIDS is often lightweight and specification-based, making it suitable for resource-constrained devices where deep packet inspection is infeasible (Mbarek, Ge & Pitner, 2020).
4. Hybrid and Distributed IDS
  Modern deployments increasingly adopt hybrid IDS architectures that combine NIDS and HIDS capabilities, often enhanced with cloud, edge, or fog computing layers (Hijazi, Alhafez & Al-khayat, 2024). Distributed IDS deploy lightweight agents across nodes while aggregating alerts at centralized or edge-based analyzers, achieving both scalability and low latency crucial for large-scale IoT ecosystems (da Silva, de Souza & Travassos, 2024; Zhukabayeva et al., 2024). While NIDS provides broad network visibility and HIDS offers deep host-level insight, neither is sufficient alone in heterogeneous and resource-constrained environments like the Internet of Things. The convergence of NIDS, HIDS, and protocol-aware PIDS supported by machine learning, edge computing, and federated analytics represents the current state-of-the-art in intrusion detection, enabling adaptive, accurate, and resilient security monitoring (Al-Garadi et al., 2020).

An intrusion detection system can be categorized by monitoring resources into Host Intrusion Detection System (HIDS) and Network Intrusion Detection System (NIDS). A HIDS operates on individual hosts or devices, analyzing system logs, file integrity, system calls, and application behavior to detect anomalies or known attack signatures at the endpoint level (Zhong et al., 2024). In contrast, a NIDS monitors network traffic by inspecting packets flowing through network segments, enabling large-scale detection of threats such as DDoS attacks, port scans, or malware propagation (Victor et al., 2023).

### Importance of intrusion detection techniques

Intrusion detection methods that rely on artificial intelligence have proven to be highly effective in overcoming many limitations of traditional approaches. These advanced techniques excel at adapting to new and evolving threats, handling dynamic network environments, and recognizing attack patterns within vast datasets (Villafranca et al., 2025). They also enable real-time identification of suspicious activity and support prompt incident response. When dealing with IoT networks, analyzing large and complex data collections is essential; however, redundant or irrelevant features in the data may unnecessarily increase the consumption of system resources, including processing time and storage requirements (Almotairi et al., 2024). Intrusion Detection Systems (IDS) are security solutions that monitor network or device activities to detect malicious actions or policy violations. In IoT, IDS is critical due to device heterogeneity, limited resources, and diverse attack surfaces. Four primary IDS paradigms dominate: Signature-based, Anomaly-based, Specification-based, and Hybrid IDS, each offering distinct detection methods tailored to evolving IoT threats (Setiawan & Soewito, 2025).

1. Signature-based IDS
  Signature-based IDS operate by matching network traffic or system behavior against a database of known attack signatures. These systems have high accuracy for detecting known threats and maintain a low false positive rate. However, they struggle to identify new or unknown attacks and require regular updates to the signature database. In IoT settings, signature-based IDS protect devices such as smart home gateways by recognizing familiar malware patterns (Elmubarak, Karrar & Hassan, 2019). Challenges arise from limited storage capacities for signatures and the overhead of frequent updates, which can burden resource-constrained IoT environments (Albulayhi et al., 2021).
2. Anomaly-based IDS
  Anomaly-based IDS detect deviations from established patterns of normal behavior in network traffic or device operations. These systems use statistical models or machine learning techniques to learn normal activity and flag anomalies as potentially malicious. Anomaly-based IDS are capable of identifying zero-day attacks and adapting to emerging threats but tend to generate higher false positive rates (Saba et al., 2022). They require extensive training data to define baselines accurately. In IoT applications like industrial networks, they monitor traffic to detect abnormal access patterns. Challenges include the variability in IoT device behavior and limited computing resources for deploying complex models (Jyothsna & Prasad, 2020).
3. Specification-based IDS
  Specification-based IDS utilize predefined rules or protocol specifications to spot behavioral deviations from allowed operations. They compare device activity against a set of rules defined by developers or administrators. This approach effectively detects unauthorized actions without needing attack signature databases and results in fewer false positives than anomaly-based systems. It’s particularly suited for strict environments, such as medical device networks, ensuring compliance with operational protocols. However, creating and maintaining detailed specifications can be resource-intensive, especially in dynamic or complex IoT environments (Amrullah, 2025).
4. Hybrid IDS
  Hybrid IDS combine signature-based and anomaly-based (or specification-based) methods to capitalize on their individual strengths as shown in Fig. 5. They typically use signature detection for known attacks and anomaly detection to find new threats, aiming to balance detection accuracy and reduce false positives (Smys, Basar & Wang, 2020). Hybrid systems are valuable in multi-layered IoT environments such as smart cities but introduce increased complexity and demand greater resources. These factors make implementation challenging in resource-constrained IoT devices, with potential issues around interoperability and system overhead (Rishiwal et al., 2025).

**Figure 5:**
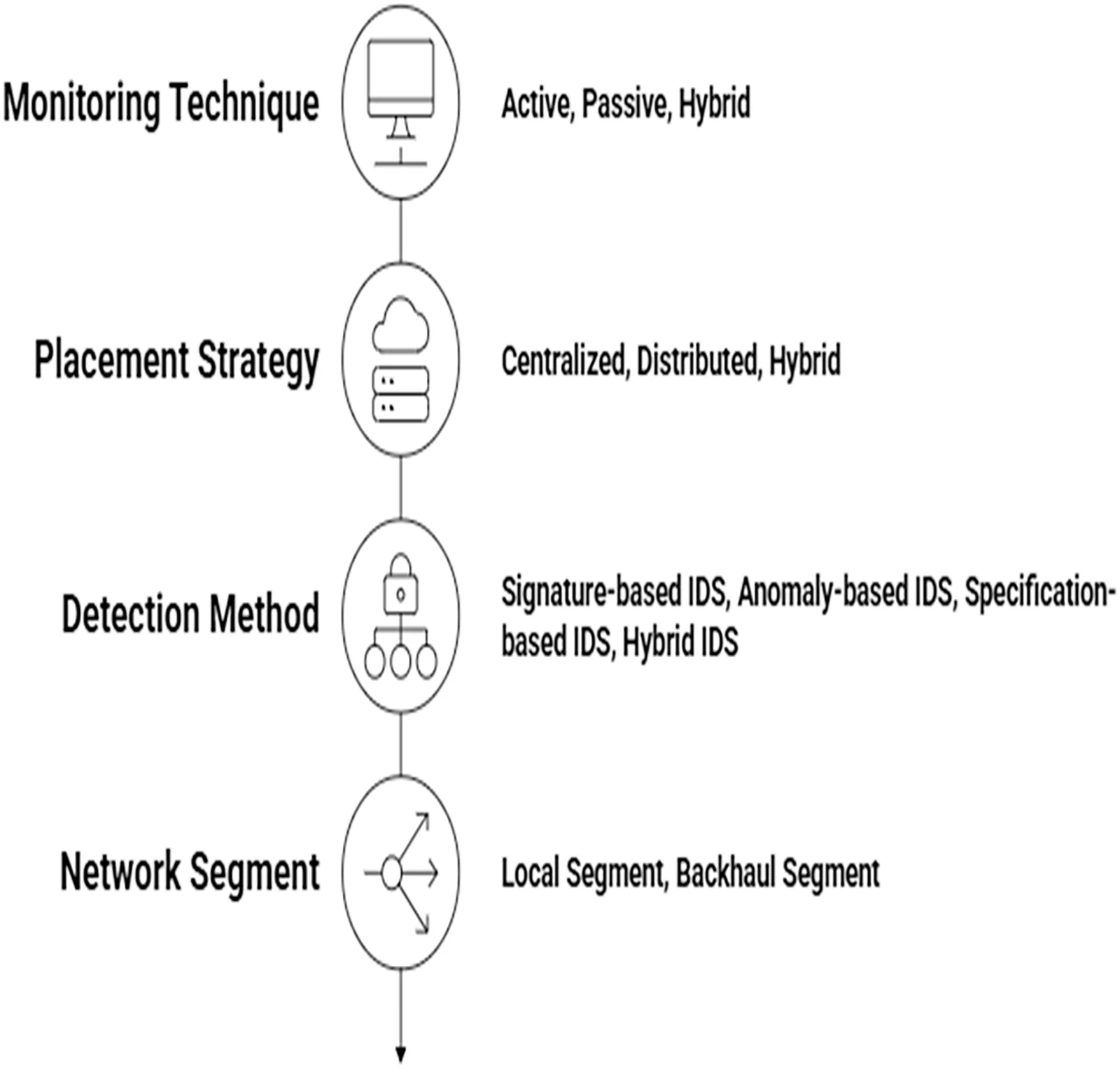
IoT network security strategies and techniques.

Placement strategies and monitoring techniques for intrusion detection systems (IDS) in IoT networks are critical to maximizing threat detection while efficiently managing resource constraints (Khraisat & Alazab, 2021). Common placement strategies include centralized IDS, where detection systems are located at a single network point to monitor overall traffic, and distributed IDS, which deploy detection modules across multiple nodes or devices to enable localized monitoring and faster response (Rahman, Al Shakil & Mustakim, 2025). Hybrid strategies combine both approaches, often clustering nodes with designated monitoring points to balance agility and oversight (Hdidou & El Alami, 2024). Monitoring techniques in IoT intrusion detection encompass both how system activity is observed and what detection logic is applied (Gyamfi & Jurcut, 2022). Observation strategies include passive monitoring (non-intrusive traffic sniffing), active monitoring (probing or injecting test traffic), and hybrid approaches that combine both to balance coverage and reactivity. These are paired with detection methodologies ranging from machine learning–based anomaly detection to specification-based approaches that enforce protocol or behavioral rules, as shown in Table 2. Crucially, the placement of monitoring components whether on-device, at the edge, in the cloud, or distributed across the network determines the quality and relevance of the collected data. Effective placement ensures that intrusion-relevant traffic is captured at optimal points, enhancing detection accuracy and reducing false positives (Perala et al., 2023). In resource-constrained IoT environments, this synergy between placement and monitoring must prioritize lightweight designs and collaborative strategies to minimize computational and energy overhead while maintaining robust, cross-layer security coverage across diverse devices and protocols (Perala et al., 2023).

**Table 2:** Summary of ID strategies and techniques in IoT.

Ref	Placement strategy			Detection method				Monitoring technique		
	Cent	Dist	Hyb	Anom	Sign	Spec	Hyb	Act	Pas	Hyb
Bagaa et al. (2020)			**✓**	**✓**						**✓**
Tripathy & Behera (2025)			**✓**			**✓**	**✓**		**✓**	
Checker, Yadav & Katarya (2023)			**✓**				**✓**			**✓**
Khraisat & Alazab (2021)	**✓**			**✓**					**✓**	
Coulibaly (2020)			**✓**				**✓**			**✓**
Khater (2021)			**✓**		**✓**				**✓**	
Kimanzi et al. (2024)			**✓**	**✓**					**✓**	
Santos et al. (2023)			**✓**			**✓**			**✓**	
Potluri (2024)			**✓**	**✓**					**✓**	
Gueriani, Kheddar & Mazari (2023)							**✓**			**✓**
Chakraborty et al. (2024)			**✓**				**✓**		**✓**	
Hijazi, Alhafez & Al-khayat (2024)		**✓**	**✓**	**✓**		**✓**			**✓**	
Manocchio et al. (2024)				**✓**					**✓**	
Göcs & Johanyák (2024)			**✓**				**✓**			**✓**
Meng et al. (2021)			**✓**				**✓**			**✓**
Holubenko et al. (2025)		**✓**		**✓**						**✓**
Huang et al. (2022)			**✓**	**✓**					**✓**	
Siddiq & Ghazwani (2024)			**✓**	**✓**					**✓**	
Ebrahimi et al. (2025)				**✓**					**✓**	
Spadaccino & Cuomo (2020)			**✓**				**✓**			**✓**
Mohammed et al. (2025)			**✓**				**✓**		**✓**	
Chen et al. (2022)			**✓**				**✓**		**✓**	
Azzaoui et al. (2024)		**✓**		**✓**						**✓**
Brooks (2022)			**✓**	**✓**					**✓**	
Protogerou et al. (2021)		**✓**	**✓**							**✓**

**Note:**

Cent, centralized; Dis, distributed; Hub, hybrid; Anom, anomaly-based; Sign, signature-based; Spec, specification-based; Act, active; Pas, passive; and Hybrid, Hyb denote monitoring techniques. **✓** indicates that the strategy or technique is supported.

Early rule-based and knowledge-based IDS heavily relied on decision tables and expert-defined rules for attack identification. The use of decision tables to verify knowledge-based systems has been advocated several times in the validation and verification literature (Yang et al., 2022; Zhong et al., 2024). However, one of the main drawbacks of these systems is that they fail to detect anomalies that occur over rule chains *i.e*., complex, multi-step attacks that span multiple rules or states and cannot be captured by isolated table entries (Gill et al., 2022; Togay et al., 2021). This limitation motivated the adoption of more advanced formal verification techniques, such as model checking, to analyze rule interactions and identify intervention policies that counter infection propagation across network states (Rezapour, GhasemiGol & Takabi, 2024; Togay et al., 2021).

In the context of the Internet of Things (IoT), both HIDS and NIDS are adapted to address resource constraints and distributed architectures, often combined in hybrid or edge-assisted deployments to achieve low-latency detection (Hindy et al., 2018). Recent advancements leverage machine learning and deep learning to enhance anomaly detection accuracy, making IDS a cornerstone of proactive defense in dynamic and heterogeneous environments (Al-Haija & Droos, 2025; Zhukabayeva et al., 2024).

## Analysis of IDS methodologies for IoT networks

The Internet of Things (IoT) has seen rapid adoption across critical domains including smart homes (Suganth & Usha, 2018), healthcare, and industrial systems (Mohamed & Aydin, 2022) heightening its attractiveness as a target for cyber threats and intensifying the demand for effective intrusion detection systems (IDS). Conventional IDS solutions are often ill-suited for IoT environments due to the unique constraints of these ecosystems, such as limited computational resources (Elrawy, Awad & Hamed, 2018), specialized communication protocols (Mohamed & Aydin, 2022; Suganth & Usha, 2018), and the need for real-time, low-overhead security mechanisms. In response, recent research has focused on developing IoT-specific IDS architectures that emphasize lightweight, efficient models capable of dynamic threat identification, reduced false positives, and resilience against evolving vulnerabilities (Bonandrini, Bercher & Zangar, 2019; Rahman, Al Shakil & Mustakim, 2025). These advancements incorporate domain-aware design principles, including protocol-specific detection logic, strategic sensor placement, and tailored security risk assessment techniques (Elrawy, Awad & Hamed, 2018; Zhukabayeva et al., 2024). Nevertheless, designing reliable, scalable, and robust IDS solutions for resource-constrained, heterogeneous IoT deployments particularly in safety-critical contexts like medical devices and smart environments remains a significant challenge (Kermani et al., 2013; Rachel, 2018).

In this study, we reviewed methodologies used in prior systematic analyses of Intrusion Detection (ID) in IoT networks. Existing reviews provide detailed insights into current detection techniques, models, performance metrics, and highlight challenges and open issues associated with machine learning (ML) and deep learning (DL) based IDS in IoT environments (Bamou et al., 2023; Thomas & Bhat, 2021). They further discuss IoT attack classifications alongside deployment and validation strategies for IDS, emphasizing the necessity for standardized evaluation metrics and the use of comprehensive, representative datasets (Singh & Singh, 2012). To refine these methodologies for analyzing the evolving threat landscape, future work should focus on developing lightweight and efficient detection models suitable for resource-constrained IoT devices, while mitigating computational complexity and reducing false positive rates (Alsoufi et al., 2021; Messaad, Jerad & Sikora, 2021). Additionally, emerging trends and new perspectives in IoT IDS must be explored for enhanced security.

In this review, four primary approaches for IDS in IoT are signature-based, anomaly-based, specification-based, and Hybrid models, as summarized in Table 3. Artificial intelligence techniques like machine learning have emerged as promising solutions due to their adaptability and self-learning capabilities, enabling dynamic threat identification, reducing false positives, and addressing evolving vulnerabilities (Zhukabayeva et al., 2024). ML-based IDS have been shown successful in detecting various attack types but face challenges such as computational complexity and high false positive rates (Arshad et al., 2020). To address these challenges, future research could focus on developing lightweight, efficient models suitable for resource-constrained IoT devices and expanding representative datasets for comprehensive evaluations (Örs & Levi, 2023). This overview underscores the critical role of selecting appropriate ML/DL models and IDS approaches tailored to IoT operational environments to maximize security effectiveness while minimizing computational costs and false alarms.

**Table 3:** Studies assessed by IoT IDS methodologies and schemes.

Scheme	ML/DL model	IoT-IDS approach	Performance
Alissa et al. (2022)	DCNN + PO	Lightweight IDS for resource-constrained devices	Evaluated on benchmark dataset (no specific metric reported)
Abdulkareem et al. (2022)	CatBoost, AdaBoost, RF	Ensemble learning classifiers (ELCs)	CatBoost achieved highest accuracy, precision, F1-score, and lowest training/testing time
Abou El Houda, Brik & Senouci (2022)	XAI (RuleFit, SHAP) + DNN	Explainable AI-based IDS	Demonstrated efficiency and model explainability
Amine, Mohamed & Denis (2021)	Nyström kernel SVM	ML-based IDS	Outperformed other ML models
Bagaa et al. (2020)	SL, NN	Anomaly-based with network pattern analysis	99.71% detection accuracy
Chakraborty et al. (2024)	DL (ReLU, Softmax) + rule-based	Hybrid IDS (DL-based anomaly detection)	99.99% accuracy for known attacks; >95% for novel attacks (CICIDS-2017)
Dutta et al. (2020)	DAE, mLSTM	Anomaly detection	Validated on IoT-23 dataset (no specific metric)
Ebrahimi et al. (2025)	1D-CNN + SHAP	Anomaly-based IDS	98.5% accuracy, 97.8% F1-score (ToN-IoT)
Göcs & Johanyák (2024)	RF, SVM (FS/Eval)	Anomaly-based IDS	RF achieved 99.1% accuracy after FS (CSE-CIC-IDS2018)
Kimanzi et al. (2024)	CNN, RNN, LSTM, autoencoders	Anomaly-based IDS	Review study; reported accuracies up to 98%+ on ToN-IoT
Manocchio et al. (2024)	Transformer (GPT/BERT variants)	Anomaly-based IDS	99.2% accuracy, 98.7% F1-score (NSL-KDD, UNSW-NB15)
Messaad, Jerad & Sikora (2021)	Federated learning	Privacy-preserving IDS	Concept validated for IoT cybersecurity (no metric reported)
Monshizadeh (2023)	Hybrid ML (SL + USL)	Hybrid anomaly detection with protocol analyzer	Scalable and effective against unknown attacks
Nagaraj & Channarayappa (2024)	RF, SVM	Hybrid IDS (detection and prevention)	99.21% accuracy on benchmark dataset
Nilsson (2020)	NN, Bayesian network, SVM	Anomaly-based detection	94% accuracy with 1% false positive rate
Nisha & Gulia (2024)	NB, DT, SVM, NN	ML-based IDS	Outperformed rule-based systems in accuracy and speed
Rahman, Al Shakil & Mustakim (2025)	SVM, RF, CNN, LSTM	Hybrid IDS	Survey study; reported performance between 95–99%
Saba et al. (2022)	CNN	Anomaly-based IDS	99.51% (NID dataset), 92.85% (BoT-IoT)
Saleem & Abdulrahman (2024)	RF, DT, HTA-GAN, CNN-LSTM, SVM	ML/DL-based IDS	Improved detection efficiency (no unified metric)
Söderström (2021)	CNN, IoTNet	Anomaly-based IDS	IoTNet did not outperform CNN; low precision observed
Tekin et al. (2023)	Decision Tree (on-device)	Energy-efficient on-device ML IDS	Reduced training/inference time and power consumption
Thulasiraman, Kaushik & Jain (2024)	RF, SVM, DT, NB, ANN	Anomaly-based IDS	98.5% accuracy after FS (UNSW-NB15)
Tripathy & Behera (2025)	SVM, RF, DT, NB, KNN	Hybrid IDS	Review study; accuracies up to 99% reported
Umair et al. (2021)	RF, KNN, SVM	Anomaly-based IDS	RF: 99.5%, SVM: 98.7%, KNN: 97.2% (NSL-KDD)
Zhukabayeva et al. (2024)	Emerging ML/DL (DL hybrids, FL)	Hybrid IDS	Review study; emerging solutions achieve 98%+
AlHamdi, Guede & Rahebi (2022)	CNN	Anomaly-based NIDS	Highly effective, particularly for DDoS detection
Alsoufi et al. (2021)	Multiple DL techniques	Hybrid and anomaly-based IDS	Supervised DL outperformed unsupervised methods
Bamou et al. (2023)	Classical ML	Distributed anomaly-based IDS	Binary and multi-class evaluation (no metric reported)
Bonandrini, Bercher & Zangar (2019)	Classical ML algorithms	Anomaly-based NIDS	Reproducible and effective results demonstrated
Chawla (2017)	ML	Hybrid IDS (signature + anomaly)	Real-time detection with low false positives
Holubenko et al. (2025)	ML	Lightweight host-based IDS	96.80% accuracy, 80% recall
Saheed et al. (2022)	Ensemble and classical ML	Anomaly-based NIDS	99.9% accuracy, 99.97% MCC
Hotellier (2024)	–	Specification-based IDS	High detection coverage, scalability, and extensibility

**Note:**

DCNN, Deep Convolutional Neural Network, XAI, Explainable Artificial Intelligence; DNN, Deep Neural Network; SL, Supervised Learning; DAE, Denoising Autoencoder; ID-CNN, Intrusion Detection Convolutional Neural Network; LSTM, Long Short-Term Memory; SHAP, SHapley Additive exPlanations; GPT, Generative Pre-trained Transformer; BERT, Bidirectional Encoder Representations from Transformers; USL, Unsupervised Learning; NB, Naïve Bayes; DT, Decision Tree; KNN, K-Nearest Neighbors.

## Benchmark datasets

The rapid expansion of the Internet of Things (IoT) has been accompanied by an urgent demand for effective intrusion detection systems capable of operating in resource-constrained, heterogeneous, and highly dynamic environments. To support the development and rigorous evaluation of machine learning and deep learning-based IDS, a rich ecosystem of publicly available IoT-specific datasets has emerged since 2018. In this study, we conducted a comprehensive review of the most widely recognized and influential benchmark datasets employed in intrusion detection system (IDS) research. Figure 6 presents a chronological timeline of key publicly available datasets spanning from 1999 to 2023, illustrating the evolution of IDS evaluation benchmarks. The upper section (1999–2016) shows the evolution of classic and early realistic network datasets (KDD Cup-99, DARPA2000, CAIDA, NSL-KDD, CTU-13, ISCX-2012, UNSW-NB15, *etc*.), whereas the lower section (2017–2023) highlights the explosive growth of IoT-specific and modern application-layer datasets (N-BaIoT, BoT-IoT, ToN-IoT, CICIDS-2017, CIC-DDoS2019, IoT-23, MedBIoT, MQTT-IoT-IDS2020, Edge-IIoTset, X-IIoTID, CICIoT2023, and others). This transition clearly illustrates the shift from traditional, often synthetic or outdated benchmarks to large-scale, real-device, protocol-aware, and multi-attack IoT-focused datasets that better reflect contemporary threat landscapes and resource-constrained environments (Alashjaee & Alqahtani, 2025; da Costa Filho et al., 2025; Ferrag et al., 2020; Hindy et al., 2018).

**Figure 6:**
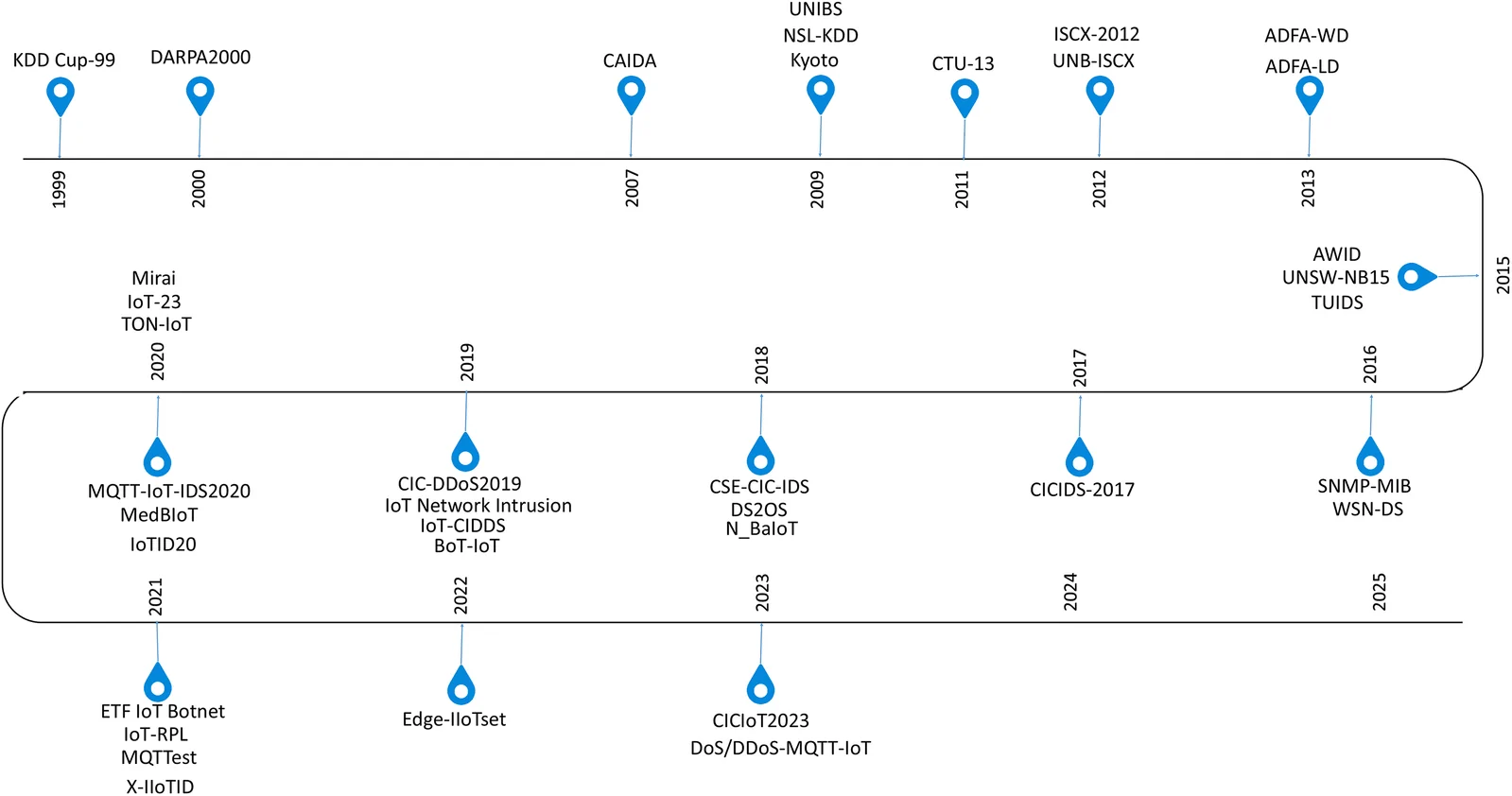
Chronological distribution of IDS datasets releases in IoT networks.

Representative benchmarks such as BoT-IoT, ToN-IoT, Edge-IIoTset, CICIoT2023, X-IIoTID, N-BaIoT, IoT-23, MQTT-IoT-IDS2020, and MedBIoT provide labeled real-world or realistic traffic from dozens to thousands of IoT devices, covering modern attack families including Mirai variants, DDoS, reconnaissance, ransomware, backdoors, and protocol-specific exploits (Al-Garadi et al., 2020; da Costa Filho et al., 2025). These datasets typically feature tens to hundreds of flow-based or statistical features, large-scale multi-class labeling (up to 34 classes in CICIoT2023), and realistic background traffic generated *via* B-Profile or actual device interaction, enabling fair comparison of anomaly-based, hybrid, and deep learning models. Recent datasets increasingly incorporate edge and fog computing scenarios, encrypted traffic, federated learning compatibility, and temporal dynamics, reflecting the evolving requirements of next-generation distributed IoT-IDS (Ferrag et al., 2020; Zhukabayeva et al., 2024). Consequently, the availability and continuous enrichment of these benchmark datasets have become a cornerstone for advancing accurate, lightweight, and explainable intrusion detection solutions tailored to the unique constraints and threats of the IoT ecosystem.

The datasets in Table 4 show a clear shift from general network benchmarks to IoT-specific datasets. Legacy datasets such as KDD Cup-99, DARPA2000, and NSL-KDD are now outdated, as they lack modern IoT protocols and realistic device communication patterns. More recent datasets like UNSW-NB15 and CICIDS-2017 improve attack coverage but still fail to capture true IoT interactions. Dataset selection involves a trade-off between realism and computational efficiency. Lightweight datasets such as DS2OS and WSN-DS are suitable for resource-constrained devices, while more realistic datasets like N-BaIoT and ToN-IoT provide richer insights but require higher computational resources. A key gap remains in protocol-specific evaluation. While datasets like MQTT-IoT-IDS2020 focus on specific protocols, they lack diversity, whereas CICIoT2023 offers broader coverage.

**Table 4:** Overview of common IDS datasets, included attacks, and key characteristics.

Dataset	Attacks included	Key characteristics
KDD Cup-99	Probe, DoS, U2R, R2L	Duplicate packets; emulated traffic; 41 features; ~5M connections; outdated but widely used ML benchmark
DARPA2000	DoS, Probe, R2L, U2R	Emulated traffic; 5-week scenario; ~4M connections; synthetic lab attacks; outdated
CAIDA	DDoS backscatter, worms, scans	Raw backbone PCAP; anonymized; unlabeled; realistic but unsuitable for supervised learning
Kyoto 2006	SSH brute-force, malware, exploits, scans, DDoS	Long-term honeypot (24 months); 24 features; session-based; highly imbalanced; 93M+ sessions
NSL-KDD	DoS, escalation, probe	Refined KDD’99; duplicates removed; 41 features; 148K records; cleaner but still outdated
UNIBS	Scans, DoS	Unlabeled real campus traffic; 3 days; PCAP; ~79K flows; anomaly detection use
CTU-13	Botnets (Neris, Rbot, NSIS.ay, Greek Bot)	13 labeled scenarios; real PCAP; ~80M packets; botnet-focused
ISCX-2012	SSH brute-force, HTTP DoS, DDoS (IRC), infiltration	Labeled PCAP; realistic background; 7 days; ~2.45M flows
UNB-ISCX (ISCX IDS)	SSH brute-force, HTTP DoS, DDoS (IRC), infiltration	Full payloads; user profiles; emulated; ~2.45M flows
ADFA-LD	Buffer overflow, Hydra-FTP/SSH, Meterpreter, Webshell	Linux system-call traces; Ubuntu 11.04; HIDS; ~10K sequences
ADFA-WD	Buffer overflow, Hydra-FTP/SSH, Java-Meterpreter, Webshell	Windows 7 system calls; HIDS; ~10K sequences
AWID	Rogue AP, de-auth, Krack, brute-force, malware, botnet	802.11 wireless PCAP; labeled; full (180M) and reduced (2.2M) versions
TUIDS	Port scans, DDoS, coordinated scans	Real-time testbed; packet and flow level; labeled; ~500K+ records
UNSW-NB15	Backdoor, DoS, exploits, fuzzers, recon, worms	Synthetic but modern; 49 features; 2.5M records; balanced classes
SNMP-MIB	DoS, brute force	SNMP MIB variables; 34 features; 4,998 records; anomaly-focused
WSN-DS	Blackhole, grayhole, flooding	Wireless sensor network dataset; 19 features; 374K records
CICIDS-2017	Brute force, botnet, DoS/DDoS, portscan, web attacks	Emulated; zero-day profiles; 80 features; ~2.8M flows
CSE-CIC-IDS	HeartBleed, DoS, botnet, brute-force, web	Real + emulated; 80 features; ~16M instances
DS2OS	Probe, DoS/DDoS, brute-force, botnet	Smart home sensors, time series, 13 features, 357K records
N-BaIoT	Mirai, BASHLITE	Real IoT traffic; 115 statistical features; 7M+ records; botnet-focused
BoT-IoT	DDoS, DoS, reconnaissance, data theft	Simulated IoT; MQTT/HTTP/DNS; 74 features; 72M+ records; highly imbalanced
CIC-DDoS2019	Multiple modern DDoS variants	Application-layer DDoS; 80+ features; ~50M flows
IoT Network Intrusion	MITM, SYN DoS, scans, Mirai	42 PCAPs; wireless IoT; ~5M+ packets
IoT-CIDDS	DoS, DDoS, brute-force, recon	Labeled IoT dataset; 47 features; 1.19M records
IoT-23	Botnet, DoS/DDoS, XSS, SQLi, portscan	Labeled Zeek logs; real malware; 23 scenarios; millions of flows
IoTID20	DoS, DDoS, recon, web attacks	Real-time IoT testbed; 86 features; 625K records
MedBIoT	Mirai, Bashlite, Torii	83 devices; PCAP; ~1M+ flows; medium-scale
MQTT-IoT-IDS2020	MQTT brute-force, aggressive scans	Simulated MQTT; PCAP + CSV; ~500K flows
TON-IoT	DoS/DDoS, ransomware, backdoor, MITM, XSS	Heterogeneous IoT; network + host logs; 22M+ records
X-IIoTID	DoS/DDoS, recon, backdoor, fuzzing	Multi-view IIoT dataset; 68 features; 820K records
Edge-IIoTset	Flooding, spoofing, malware, injection	Edge-focused IoT; 15–23 features; 58K records
CICIoT2023	33 attacks including Mirai, spoofing, DDoS	Real-time 105-device IoT; PCAP/CSV; 47 features; ~46M records

## Analysis of key findings in IoT-IDS

Selecting the right IDS paradigm for IoT depends largely on the device capabilities, threat profile, and application environment. While signature-based IDS excels in detecting known threats efficiently, anomaly-based approaches offer adaptability to new attacks albeit with more false positives. Specification-based IDS suits environments where strict behavior control is feasible, whereas hybrid IDS offers the most comprehensive security, though at the cost of complexity (Da Costa et al., 2019). The results unequivocally demonstrate that neural-network-centric approaches (NN, CNN, LSTM) now constitute the majority of state-of-the-art IoT-IDS research as illustrated in Fig. 7, and reflecting their exceptional ability to model the high-dimensional, non-linear, and temporal patterns inherent in modern IoT traffic and host logs, while classical yet robust algorithms (SVM, RF, DT) continue to play a critical complementary role in hybrid (Al-Hameli et al., 2021), lightweight, and explainable designs. This dual trend perfectly mirrors the core challenge of IoT security: achieving near-perfect detection accuracy against evolving threats while respecting severe computational, energy, and real-time constraints.

**Figure 7:**
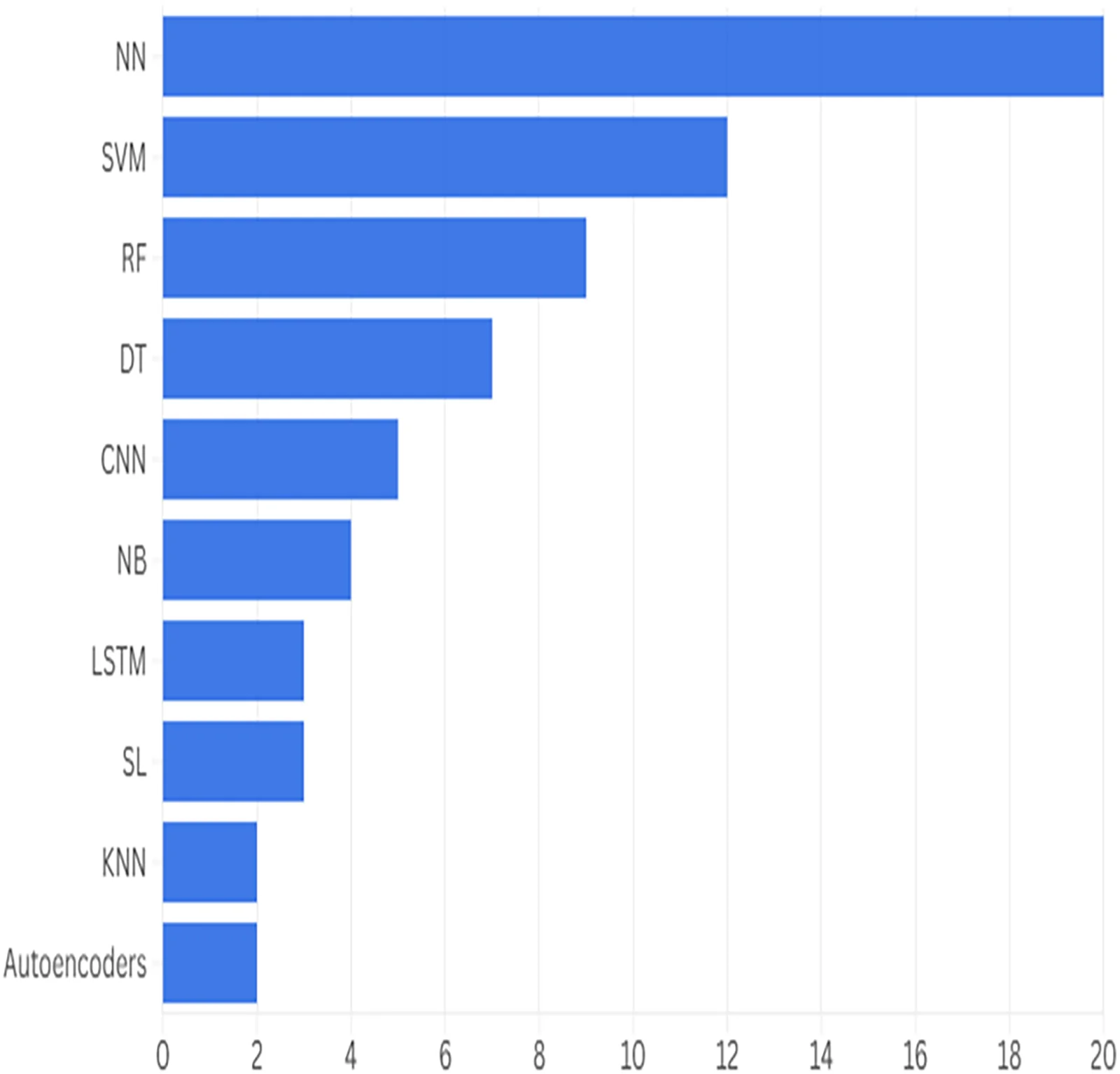
Top 10 common ML/DL model implemented in IoT-based IDS.

The heatmap in Fig. 8 illustrates the variation in accuracy across different ML/DL models used in IoT intrusion detection systems, highlighting that most approaches achieve high performance above 98%. Notably, hybrid and deep learning-based models, such as DL with rule-based methods and CNN variants, demonstrate the highest accuracy, indicating their effectiveness in detecting complex cyber threats.

**Figure 8:**
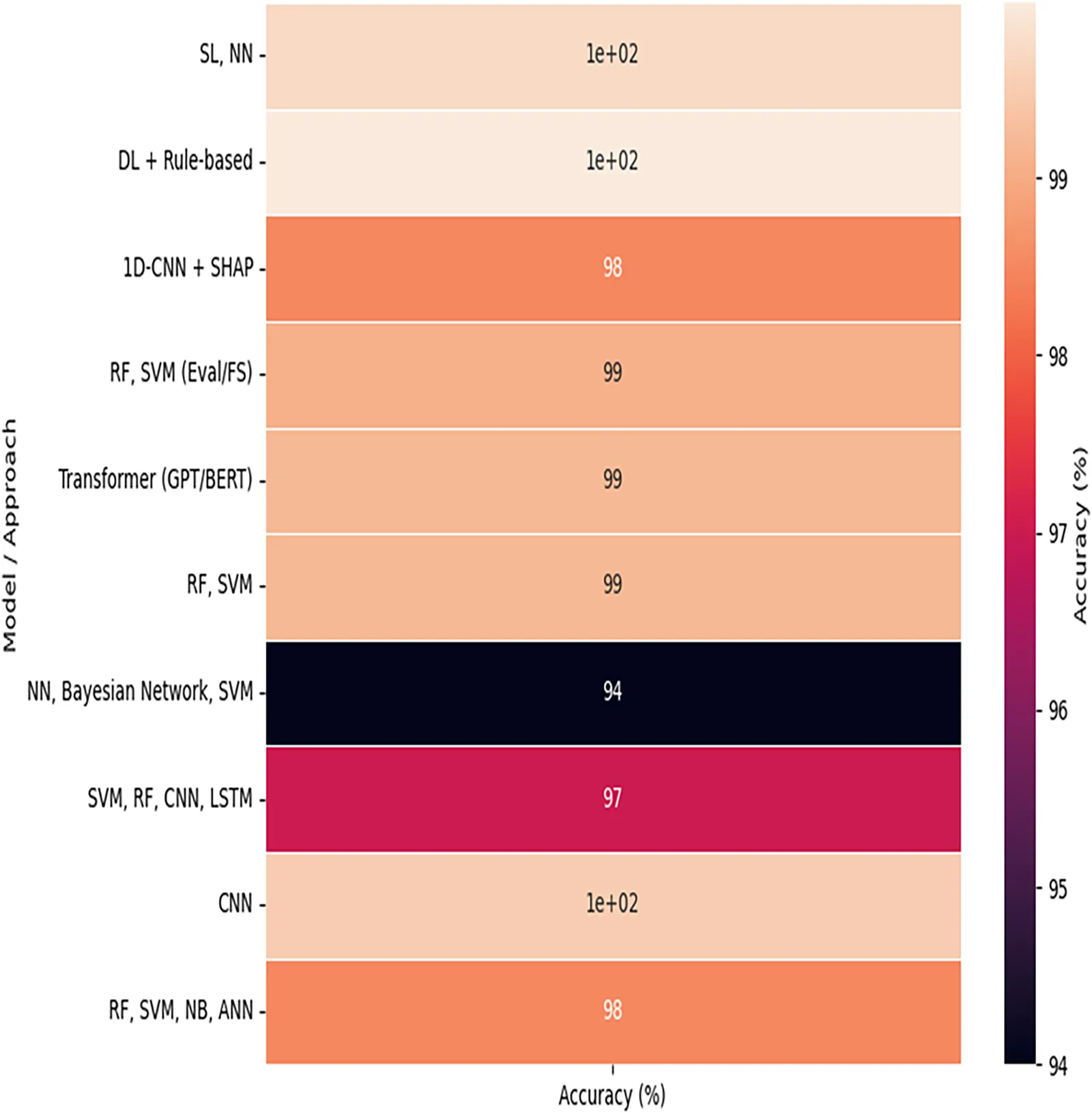
Heatmap of ML/DL model accuracy in IoT IDS.

The overwhelming dominance of anomaly-based approaches (58% of recent publications) clearly reflects the research community’s consensus that IoT networks predominantly face zero-day, polymorphic, and insider threats that cannot be countered by static signatures. Deep learning and statistical anomaly detection have become the *de facto* standard because they can learn normal behavior from the highly heterogeneous and dynamic traffic generated by resource-constrained devices, smart objects, and diverse protocols (MQTT, CoAP, RPL, *etc*.). The strong presence of hybrid IDS (31%) demonstrates a maturing trend: researchers increasingly combine the low false-positive rate of signature/specification methods for known attacks with the adaptability of anomaly detection for unknown threats, achieving detection rates frequently exceeding 98% while maintaining interpretability and lower resource overhead in edge-assisted deployments. This aligns with findings reported in recent studies (Abou El Houda, Brik & Senouci, 2022; Zhukabayeva et al., 2024). The relatively small share of pure classical ML (11%) and signature/specification-only IDS (8%) confirms that standalone traditional methods are now considered insufficient for modern IoT threat landscapes. Signature-based systems, while still lightweight and deterministic, fail against the rapid evolution of IoT botnets (Mirai variants, MooBot, *etc*.) and application-layer attacks. Similarly, non-deep classical ML models struggle with the high-dimensional, temporal, and encrypted traffic typical of current IoT ecosystems.

The distribution shown in Fig. 9 validates that anomaly-based and hybrid paradigms have become the cornerstone of state-of-the-art IoT intrusion detection systems, offering the best trade-off between detection capability, adaptability to novel threats, and feasibility on resource-constrained and distributed architectures.

**Figure 9:**
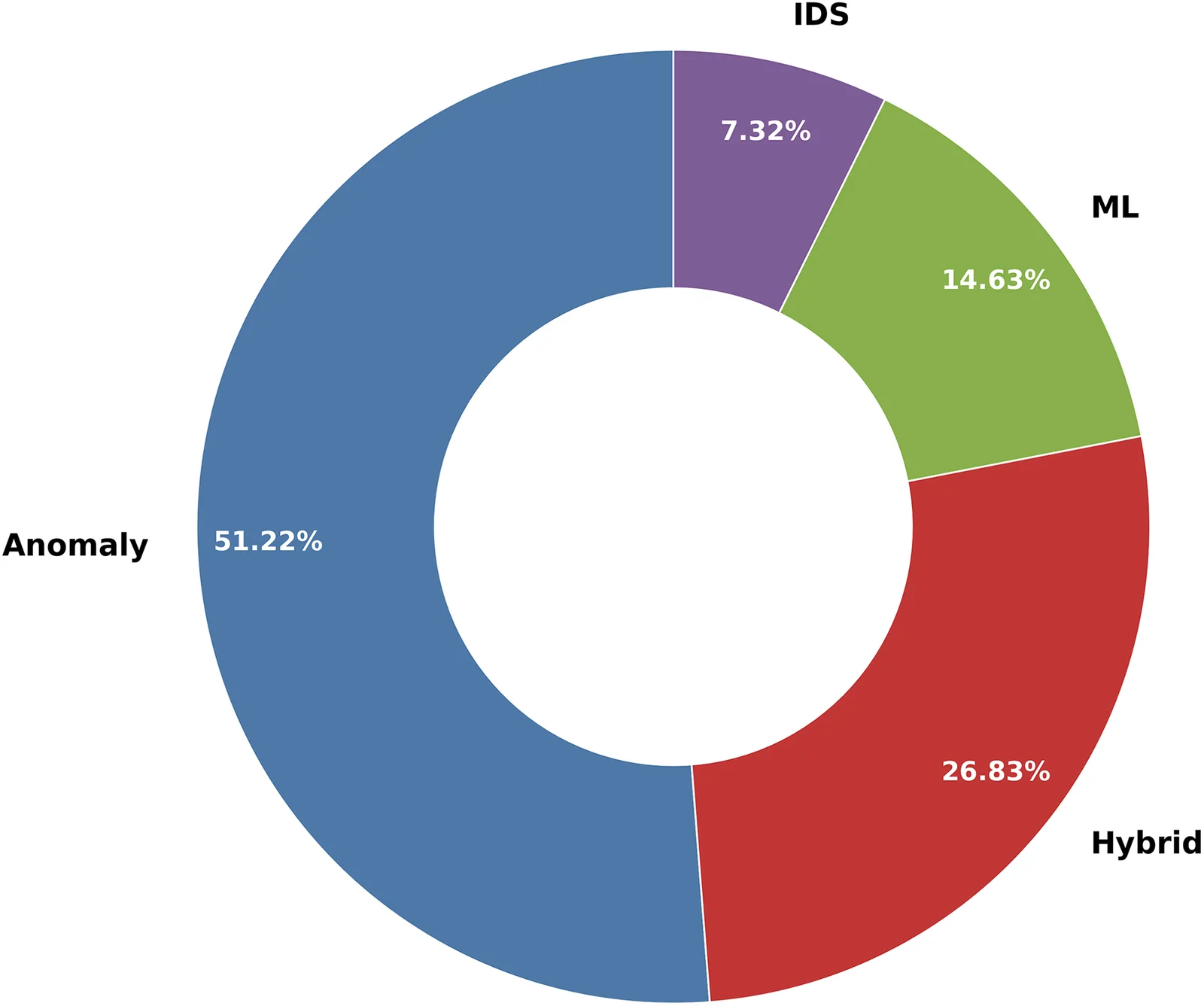
Most used ID techniques for IoT networks.

## Optimization strategies and model correlations in IoT-IDS

This section is to a targeted analysis of how optimization strategies are systematically mapped to ML/DL models to overcome the inherent constraints of IoT environments. The integration of optimization is not merely an auxiliary step but a fundamental requirement for deploying IDS on resource-constrained hardware. By examining the interplay between optimization algorithms such as meta-heuristic feature selection, parameter pruning, and lightweight architectural design and their corresponding learning models, we uncover specific correlations that dictate the feasibility of an IDS. The following analysis evaluates how these correlations specifically enhance detection accuracy while minimizing the computational overhead, energy consumption, and latency that typically hinder standard ML/DL applications in the IoT ecosystem. As evidenced in Table 5, a strong correlation exists between Feature Selection (FS) and Classical ML models (RF/SVM), which consistently yields accuracies above 98% while significantly lowering the memory footprint. Conversely, for Deep Learning (CNN/LSTM), the correlation shifts toward Architectural Optimization (Lightweighting) and XAI-driven pruning. This analysis reveals that while DL models provide superior detection for complex patterns, they require specific optimization such as the PO-optimized DCNN used by Alissa et al. (2022) to remain feasible for resource-constrained edge devices.

**Table 5:** Comparative framework of optimization techniques and ML/DL model correlations.

Optimization strategy	Specific technique	Correlated ML/DL model	Key outcome/impact	IoT constraint addressed	Supporting study
Feature selection (FS)	Heuristic/statistical FS	RF, SVM, NB, ANN	99.1% accuracy; reduced input dimensionality	Memory and processing power	Göcs & Johanyák (2024), Thulasiraman, Kaushik & Jain (2024)
Architectural optimization	Lightweight DCNN/IoTNet	DCNN, CNN	High efficiency on-device; IoTNet optimized for edge	Battery life and latency	Alissa et al. (2022), Söderström (2021)
Model lightweighting	On-device DT/ensemble	Decision trees, CatBoost	Reduced inference time; lowest training/testing overhead	Real-time applicability	Tekin et al. (2023), Abdulkareem et al. (2022)
Interpretability and pruning	XAI (SHAP, RuleFit)	DNN, 1D-CNN	Identified critical features to prune redundant neurons	Model complexity	Abou El Houda, Brik & Senouci (2022), Ebrahimi et al. (2025)
Algorithmic hybridization	Hybrid DL + rule-based	CNN-LSTM, GAN	99.9% accuracy for known attacks; improved efficiency	Detection reliability	Chakraborty et al. (2024), Saleem & Abdulrahman (2024)
Decentralized optimization	Federated learning (FL)	Distributed hybrids	Privacy-preserved detection without raw data transfer	Bandwidth and privacy	Messaad, Jerad & Sikora (2021), Zhukabayeva et al. (2024)

## Analysis challenges in intrusion detection for IoT

IoT systems often lack sufficient protection against network attacks due to the severe processing and energy restrictions that do not allow the use of standard Intrusion Detection Systems (IDS) on IoT devices (Kim, Kim & Park, 2020). However, IDSs have been successfully employed in similar network types such as Mobile Adhoc Networks (MANET), Wireless Sensor Networks (WSN), and Cyber Physical Systems (CPS), and research is ongoing to develop IDSs better suited to the limitations of IoT devices (Bonandrini, Bercher & Zangar, 2019). Machine learning approaches, such as artificial intelligence, offer potential solutions for enhancing intrusion detection in IoT environments by enabling dynamic threat identification and reducing false positives (Sarker, 2023). Nevertheless, computational complexity and high false positive rates remain significant challenges in implementing ML and DL-based IDS for IoT networks (Bishop, 2006). Many Internets of Things (IoT) systems run on tiny connected devices that have to deal with severe processor and energy restrictions (Kim, Kim & Park, 2020).

The literature review in Table 5 synthesizes methodological approaches and key challenges identified across multiple systematic reviews and studies on intrusion detection in IoT networks. A recurring theme is the lack of standardization, with many works highlight the absence of common evaluation metrics, benchmark datasets, and reproducible protocols, which hinders fair comparison and real-world validation. Many studies rely heavily on synthetic or single datasets, limiting generalizability across diverse IoT domains and network topologies. Additionally, current IDS taxonomies and models often fail to address dynamic, evolving threats, such as zero-day attacks, AI-powered evasion, LLM-based exploits, and cross-layer attack chains. There is also a notable gap in practical deployment considerations: insufficient attention to protocol-specific vulnerabilities (*e.g*., CoAP, MQTT), resource constraints in edge/fog environments, privacy-preserving mechanisms like federated learning, and scalability under real-world loads. Collectively, these gaps underscore the need for adaptive, empirically validated, and context-aware IDS frameworks that bridge the divide between theoretical models and the heterogeneous, resource-constrained reality of IoT ecosystems.

The performance of Intrusion Detection Systems (IDS) in Internet of Things (IoT) environments depends on the network topology (Arshad et al., 2019; Kalnoor & Gowrishankar, 2021). Limited processing resources in IoT devices restrict the use of standard security mechanisms, making lightweight IDS solutions crucial (Amine, Mohamed & Denis, 2021). Machine learning approaches, such as artificial intelligence and deep learning, offer dynamic threat identification and reduced false positives in IoT IDSs (Heorhiadi, Reiter & Sekar, 2012). However, computational complexity and high false positive rates remain challenges in ML/DL-based IDSs for IoT. The heterogeneity and diversity of IoT data sets are crucial for effective detection performance and standardized evaluation metrics (Beremark & Fryland, 2018). Advancements in architectural designs and second line defenses are essential for securing IoT networks and developing efficient IDS solutions (Arshad et al., 2019).

While the 16 reviews in Table 6 highlight key challenges such as the lack of standardized metrics and limited consideration of edge constraints, they mainly identify problems without explaining how optimization strategies address them in practice. This reveals an “Optimization-Deployment Gap” in current literature. This study provides three key insights. First, optimization is not universal; its effectiveness depends on the model type, emphasizing the need for architecture-aware optimization. Second, focusing solely on accuracy creates a “metric paradox,” as highly accurate models may be too complex for real-time IoT use, motivating the need for efficiency-aware metrics. Third, existing approaches rely on static optimization, whereas future IoT-IDS must adopt adaptive optimization to handle dynamic environments and concept drift.

**Table 6:** Review of IDS challenges identified for IoT network by recent studies.

Ref.	Review type and scope	Systematic methodology used	Challenges identified
Khraisat & Alazab (2021)	Survey of contemporary IDS techniques	Literature analysis; dataset evaluation (ToN-IoT, BoT-IoT); benchmarking (accuracy, FPR, latency); challenge mapping	Lack of standardized evaluation metrics; overreliance on synthetic datasets; weak protection against zero-day and evolving threats
Mikuláš & Kotuliak (2024)	Taxonomy-based comprehensive review of IoT IDS	Structured taxonomy of attack types, detection techniques, deployment strategies, and validation methods	Static taxonomy limits adaptivity; insufficient discussion of AI-driven evasion and LLM-based threats
Aparcana-Tasayco, Deng & Park (2025)	Architectural and practice-oriented review	Evaluation of IDS suitability across centralized, distributed, and fog architectures	Limited empirical validation; minimal focus on protocol-layer vulnerabilities (*e.g*., CoAP, MQTT)
Mallidi & Ramisetty (2025)	ML/DL-focused IoT IDS review	Mapping of ML/DL models to datasets; identification of generalizability gaps; future research framework	No consideration of concept drift; ignores federated and privacy-preserving learning constraints
Sasi et al. (2024)	Taxonomy-driven survey of IoT attacks and IDS	Classification of physical, network, and application-layer attacks; review of mitigation techniques	Limited discussion of cross-layer attacks; absence of structured evidence-quality assessment (*e.g*., PRISMA)
Alsoufi et al. (2021)	Systematic literature review on DL-based anomaly IDS	PRISMA-compliant SLR; five databases searched; 26 studies selected; analysis of seven DL techniques	Focus restricted to anomaly detection; no cross-domain comparison
Thomas & Bhat (2021)	Review of ML/DL techniques for IDS	Database search (IEEE, ScienceDirect); focus on algorithm–dataset pairing	No temporal trend analysis; neglects edge and cloud deployment constraints
Bonandrini, Bercher & Zangar (2019)	Empirical IDS evaluation study	Reproducible ML experiments using CIDDS-001 dataset	Single dataset limits generalizability; no topology or protocol diversity
Bamou et al. (2023)	Comprehensive review with empirical validation	Review of algorithms, datasets, and applications; experimental testing of selected models	Absence of standardized evaluation protocols; limited IoT-specific attack coverage
Messaad, Jerad & Sikora (2021)	Survey of AI/ML/DL for IoT IDS	Literature survey with emphasis on federated learning	No empirical validation; shallow treatment of adversarial AI threats
Satam (2019)	Protocol-specific micro-IDS design study	Threat modeling; protocol fingerprinting; ML training per protocol	Scalability issues across multiple protocols; lack of cross-protocol correlation
Raheema (2023)	DL-based DDoS detection study	Sparse convolutional network with evolutionary optimization	Narrow focus on DDoS; no comparison with alternative ML/DL approaches
Haque et al. (2023)	Systematic review of ML/DL trends and datasets	Analysis of IoT datasets; comparison of ML/DL usage trends and efficiencies	No temporal evolution of threats; lacks threat actor motivation analysis
Zhukabayeva et al. (2024)	Broad review of challenges and emerging solutions	Review of ML, FL, blockchain, GANs, and RL for IoT security	No empirical validation; unclear implementation roadmaps
Mills et al. (2021)	Framework for emerging IoT threats (Citrus)	Anomaly detection integrated with CTI, graph metrics, and ML	Limited scalability evaluation; absence of open-source implementation
Gyamfi & Jurcut (2022)	MEC-enabled IoT IDS review	Comparative analysis of datasets, metrics, and deployment strategies; MEC-based NIDS framework	No assessment of MEC reliability under load; privacy concerns at the edge ignored

## Analysis of recent studies

To build upon and extend prior findings, this study includes an updated systematic literature review encompassing all relevant works published from January 2020 to November 2025, with a strong emphasis on the latest and most rapidly developing research. The update adhered strictly to the original review protocol—employing the same search strategy, inclusion and exclusion criteria, and reliance on Scopus, IEEE Xplore, Web of Science, along with supplementary sources. After deduplication, 118 unique peer-reviewed journal and conference articles were identified and thoroughly analyzed.

As shown in Fig. 10, the annual publication count reveals a consistent and accelerating growth trajectory: starting with 15 articles in 2020, rising to 17 in 2021, and reaching 21 publications already in 2025 (as of November). This upward trend underscores the sustained and intensifying scholarly interest in intrusion detection for IoT, reflecting the field’s dynamic evolution and increasing relevance in contemporary cybersecurity research.

**Figure 10:**
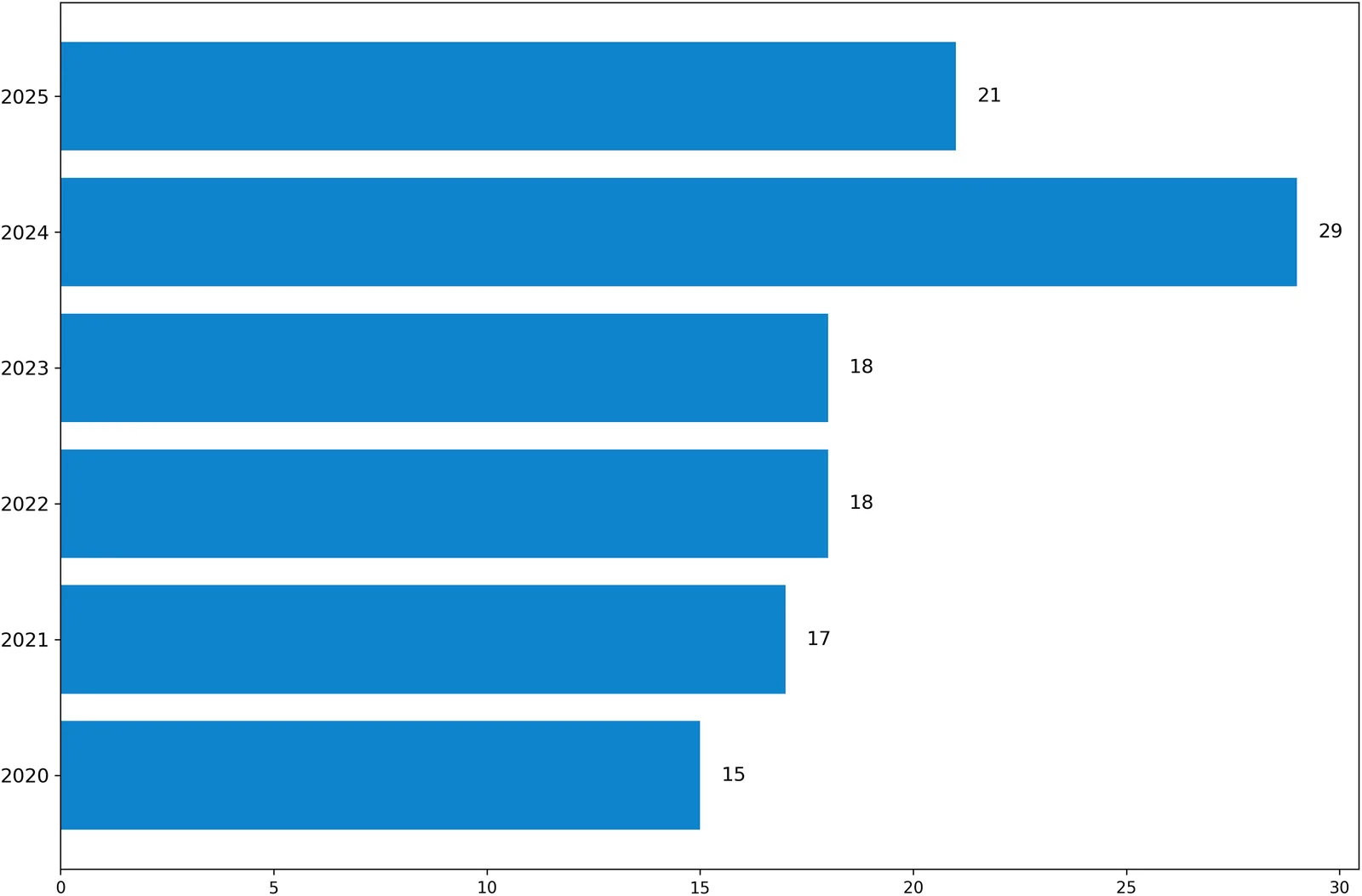
Publications by year (2020–2025).

Figure 11 further illustrates the distribution of the 118 publications from 2020 to 2025 by publication type and indexing source. Journal articles constitute the majority (approximately 82%), underscoring the growing maturity of the field, while conference articles account for about 20%. The remaining 16% comprises other sources, including book chapters, theses, and technical reports.

**Figure 11:**
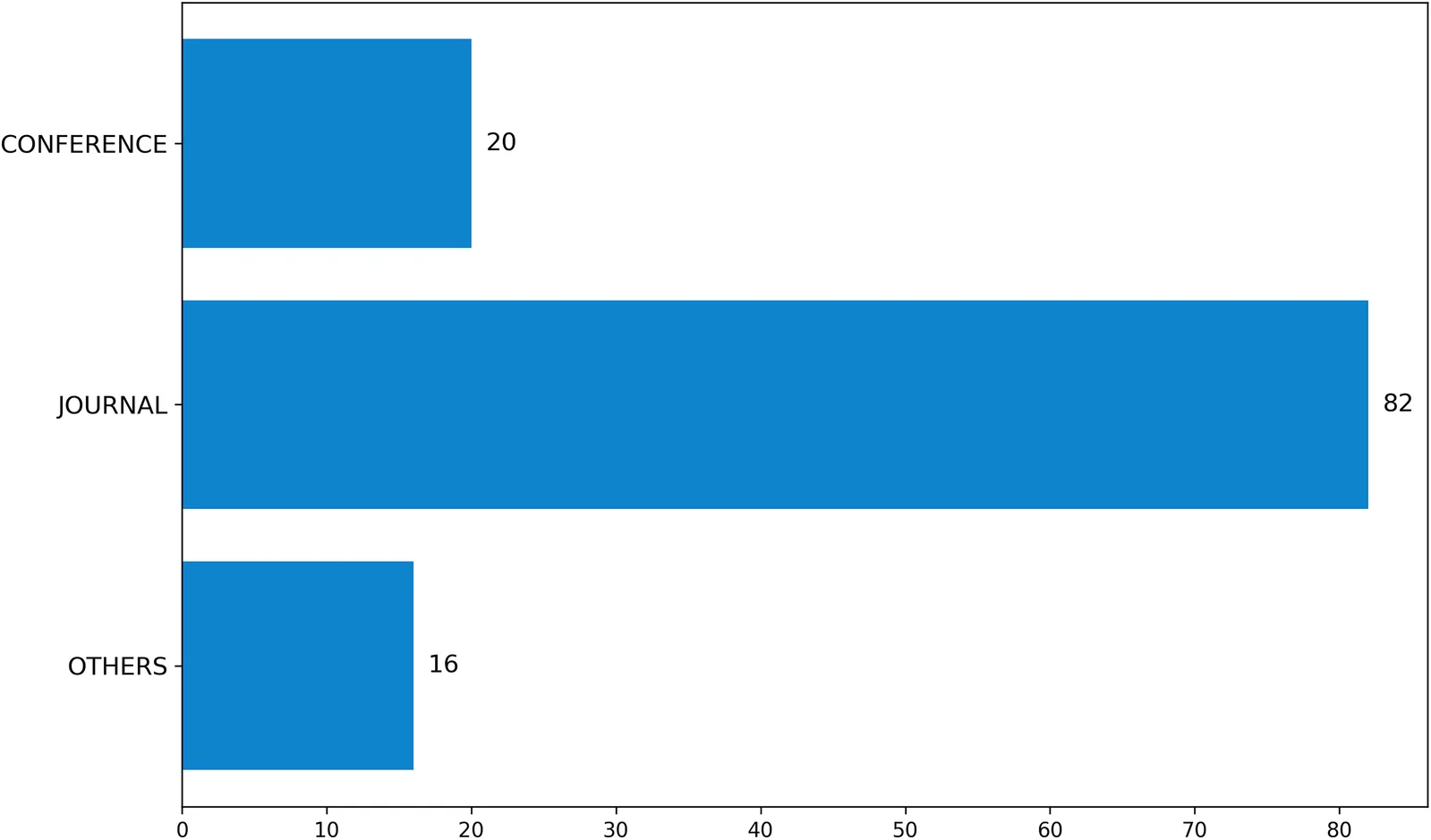
Published articles distribution between 2020–2025.

As illustrated in Fig. 12, the distribution of the selected studies across indexing databases is as follows:

- Web of Science (WoS) covers the largest share at approximately 42%, with strong representation in high-impact journals.
- Scopus indexes 25.5% of the studies, affirming its role as a major and comprehensive source for this research domain.
- IEEE Xplore accounts for 19% of the publications, primarily comprising IEEE-sponsored journals and conference proceedings.
- The remaining 14% consists of works not indexed in Scopus, IEEE Xplore, or Web of Science, including preprints, theses, and publications from other or non-traditional venues.

**Figure 12:**
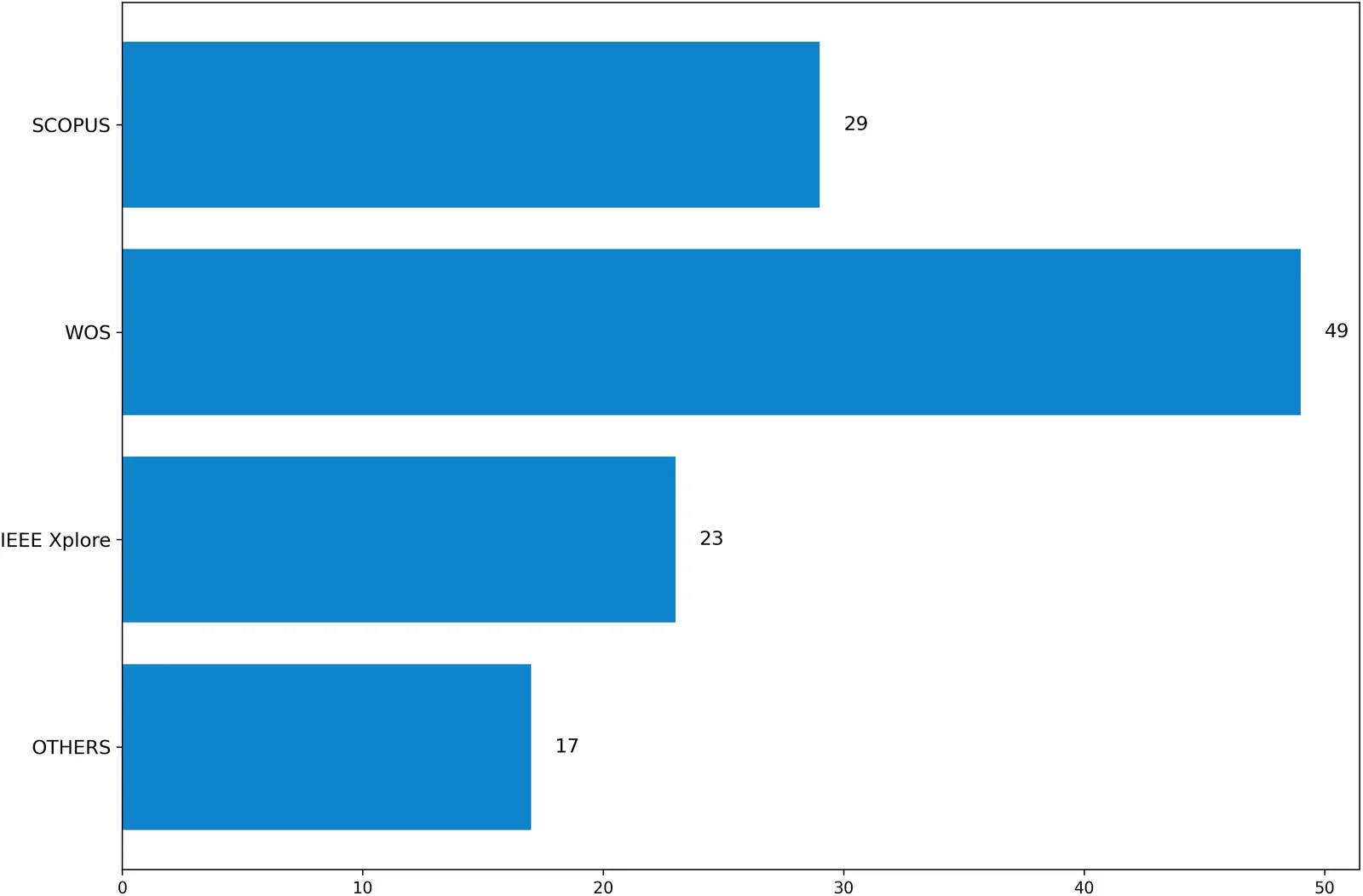
Classification articles per between 2020–2025.

Journal articles are especially prevalent in Scopus-indexed venues (MDPI, Elsevier, Springer, Wiley, and Taylor & Francis), whereas IEEE Xplore remains the primary source for IEEE-branded journals and conferences. The significant overlap between Scopus and WoS underscores the high academic quality of the selected works. This updated distribution provides a robust and current view of the research landscape, highlighting both the increasing volume and the sustained publication of high-quality, peer-reviewed contributions in the field of IoT intrusion detection.

## Deployment recommendations

The placement of IDS in IoT networks affects detection efficiency, scalability, and responsiveness.

– Centralized deployment: All traffic is analyzed at a single node, simplifying management but potentially creating bottlenecks and latency issues.
– Distributed deployment: Detection agents are placed near IoT devices, improving real-time monitoring, fault tolerance, and scalability.
– Hybrid deployment: Combines both approaches, with local agents detecting anomalies and a central node coordinating analysis and responses, balancing accuracy and efficiency.

Importance of IoT-IDS Approach: Selecting an appropriate IDS signature-based, anomaly-based, or hybrid should align with the deployment strategy. Proper placement enhances detection accuracy, reduces false positives, and ensures timely threat mitigation in heterogeneous IoT networks.

## Future directions

Future IoT IDS research must overcome key structural and methodological limitations to enable scalable, resilient, and real-world deployment. First, the field needs standardized benchmarking frameworks supported by diverse, realistic, multi-modal IoT datasets that reflect modern environments and enable fair, reproducible evaluation. Second, IDS models should shift from isolated, network-centric approaches to cross-layer architectures that integrate physical-layer signals, protocol behaviors, and application-level semantics to detect complex cyber–physical attacks. Third, adversarial robustness must become a core requirement, with IDS evaluated against evasion, poisoning, and model-extraction attacks while incorporating certified defenses. Fourth, privacy-preserving mechanisms such as federated learning, encryption, and explainable, auditable outputs are essential for compliance with regulations in sensitive domains. Finally, the field should progress toward autonomous, self-healing security systems that integrate with SDN, zero-trust, and automated orchestration to enable proactive, closed-loop response.

Collectively, these directions pave the way for trustworthy, adaptive, and regulation-aligned IoT IDS capable of protecting diverse and resource-constrained environments. Looking ahead, IoT IDS research is expected to focus on lightweight, adaptive hybrid models incorporating advanced AI and edge computing to address IoT’s unique constraints. Enhanced collaboration among devices and standardized security frameworks will further advance IDS effectiveness in safeguarding the expanding IoT ecosystem as of 2025.

## Conclusion

This study provides a systematic review of IoT intrusion detection systems with a focus on optimization-driven ML/DL approaches. The findings show that techniques such as feature selection, hyperparameter tuning, and model lightweighting are essential for improving detection accuracy while meeting IoT constraints like limited resources, energy efficiency, and real-time operation. The analysis highlights that optimization is model-dependent, emphasizing the need for architecture-aware strategies and revealing clear correlations between optimization methods and ML/DL models. It also identifies an optimization-deployment gap, where high performance on benchmark datasets does not translate well to real-world IoT environments, partly due to unrealistic datasets and overreliance on accuracy as the main metric. Future work should focus on adaptive and lightweight optimization techniques, efficiency-aware evaluation metrics, and realistic IoT datasets. This ensures better alignment between model performance and practical deployment, supporting the development of effective and scalable IoT-IDS solutions.

## Supplemental Information

10.7717/peerj-cs.4048/supp-1 Supplemental Information 1 datasets info.

## Additional Information and Declarations

### Data Availability

The following information was supplied regarding data availability:

This is a Literature Review.

## References

[ref-1] Abdulkareem SA, Foh CH, Lee H, Carrez F, Moessner K (2022). IoT network intrusion detection with ensemble learners.

[ref-2] Abou El Houda Z, Brik B, Senouci S-M (2022). A novel IoT-based explainable deep learning framework for intrusion detection systems. IEEE Internet of Things Magazine.

[ref-3] Al-Garadi MA, Mohamed A, Al-Ali AK, Du X, Ali I, Guizani M (2020). A survey of machine and deep learning methods for Internet of Things (IoT) security. IEEE Communications Surveys & Tutorials.

[ref-4] Al-Haija QA, Droos A (2025). A comprehensive survey on deep learning-based intrusion detection systems in internet of things (IoT). Expert Systems.

[ref-5] Al-Hameli BA, Alsewari A, Alraddadi AS, Aldhaqm A (2021). Classification algorithms and feature selection techniques for a hybrid diabetes detection system. International Journal of Computational Intelligence in Control.

[ref-6] Alam H, Tomai E (2023). Security attacks and countermeasures in smart homes. International Journal on Cybernetics & Informatics (IJCI).

[ref-7] Alashjaee AM, Alqahtani F (2025). Enhanced intrusion detection system IoT network security model by feed forward neural network and machine learning. Scientific Reports.

[ref-8] Albulayhi K, Smadi AA, Sheldon FT, Abercrombie RK (2021). IoT intrusion detection taxonomy, reference architecture, and analyses. Sensors.

[ref-9] AlHamdi MJM, Guede JML, Rahebi J (2022). Convolutional neural network for malware detection in IoT network.

[ref-10] Alissa AK, Alrayes SF, Tarmissi K, Yafoz A, Alsini R, Alghushairy O, Othman M, Motwakel A (2022). Planet optimization with deep convolutional neural network for lightweight intrusion detection in resource-constrained IoT networks. Applied Sciences.

[ref-11] Almotairi A, Atawneh S, Khashan OA, Khafajah NM (2024). Enhancing intrusion detection in IoT networks using machine learning-based feature selection and ensemble models. Systems Science & Control Engineering.

[ref-12] Alsoufi MA, Razak S, Siraj MM, Nafea I, Ghaleb FA, Saeed F, Nasser M (2021). Anomaly-based intrusion detection systems in IoT using deep learning: a systematic literature review. Applied Sciences.

[ref-13] Amine K, Mohamed H, Denis H (2021). Machine learning based intrusion detection for cyber-security in IoT networks.

[ref-14] AMR (2025). IT threat evolution in Q1 2025: non-mobile statistics.

[ref-15] Amrullah A (2025). A review and comparative analysis of intrusion detection systems for edge networks in IoT. Intellithings Journal.

[ref-16] Aparcana-Tasayco AJ, Deng X, Park JH (2025). A systematic review of anomaly detection in IoT security: towards quantum machine learning approach. EPJ Quantum Technology.

[ref-17] Arshad J, Azad MA, Amad R, Salah K, Alazab M, Iqbal R (2020). A review of performance, energy and privacy of intrusion detection systems for IoT. Electronics.

[ref-18] Arshad J, Azad MA, Mahmoud Abdellatif M, Ur Rehman MH, Salah K (2019). COLIDE: a collaborative intrusion detection framework for Internet of Things. IET Networks.

[ref-19] Azzaoui H, Boukhamla AZE, Perazzo P, Alazab M, Ravi V (2024). A lightweight cooperative intrusion detection system for RPL-based IoT. Wireless Personal Communications.

[ref-20] Bagaa M, Taleb T, Bernabe JB, Skarmeta A (2020). A machine learning security framework for IoT systems. IEEE Access.

[ref-21] Bamou A, El Ouadghiri MD, Aghoutane B, Maada L (2023). IDS based on machine learning in reaction to IoT attacks: review and empirical evaluation. International Journal on Advanced Science, Engineering & Information Technology.

[ref-22] Beremark M, Fryland J (2018). A comparison of intrusion detection systems in home networks. Information Security Technical Report.

[ref-23] Bishop CM (2006). Pattern recognition and machine learning. Journal of Electronic Imaging.

[ref-24] Bonandrini V, Bercher J-F, Zangar N (2019). Machine learning methods for anomaly detection in IoT networks, with illustrations.

[ref-25] Brooks T (2022). The professionalization of the hacker industry.

[ref-26] Castro OEL, Deng X, Park JH (2023). Comprehensive survey on AI-based technologies for enhancing IoT privacy and security: trends, challenges, and solutions. Human-Centric Computing and Information Sciences.

[ref-27] Celdrán AH, Sánchez PMS, Castillo MA, Bovet G, Pérez GM, Stiller B (2023). Intelligent and behavioral-based detection of malware in IoT spectrum sensors. International Journal of Information Security.

[ref-28] Ch MSS, Yamarthi NR (2025). Design of an iterative method leveraging deep Q-networks for intrusion detection system operations. IEEE Access.

[ref-29] Chakraborty S, Pandey SK, Maity S, Dey L (2024). Detection and classification of novel attacks and anomaly in IoT network using rule based deep learning model. SN Computer Science.

[ref-30] Chawla S (2017). Deep learning based intrusion detection system for Internet of Things.

[ref-31] Checker S, Yadav M, Katarya R (2023). A survey on intrusion detection system for IoT networks based on artificial intelligence.

[ref-32] Chen Z, Liu J, Shen Y, Simsek M, Kantarci B, Mouftah HT, Djukic P (2022). Machine learning-enabled IoT security: open issues and challenges under advanced persistent threats. ACM Computing Surveys.

[ref-33] Cheng J, Pang H, Yan H, Zhang D (2025). Protocol-based PID control of switched systems with DoS attacks. IEEE Transactions on Information Forensics and Security.

[ref-34] Chua T-H, Salam I (2022). Evaluation of machine learning algorithms in network-based intrusion detection system.

[ref-35] Coulibaly K (2020). An overview of intrusion detection and prevention systems.

[ref-36] Cui M, Han D, Liu H, Li K-C, Tang M, Chang C-C, Ayaz F, Sheng Z, Guan YL (2024). Secure data sharing for consortium blockchain-enabled vehicular social networks. IEEE Transactions on Vehicular Technology.

[ref-37] da Costa Filho PE, Marques LAdA, Lima IdSFd, Sousa ELd, Kreutz ME, Neto AV, Cunha EN, Vieira D (2025). Machine-learning-based classification of electronic devices using an IoT smart meter. Informatics.

[ref-38] Da Costa KAP, Papa JP, Lisboa CO, Munoz R, de Albuquerque VHC (2019). Internet of Things: a survey on machine learning-based intrusion detection approaches. Computer Networks.

[ref-39] da Silva FNR, de Souza BP, Travassos GH (2024). A literature study on application domains and IoT software systems architectures solutions influencing quality requirements.

[ref-40] de Souza CA, Westphall CB, Machado RB, Loffi L, Westphall CM, Geronimo GA (2022). Intrusion detection and prevention in fog based IoT environments: a systematic literature review. Computer Networks.

[ref-41] Deogirikar J, Vidhate A (2017). Security attacks in IoT: a survey.

[ref-42] Drewek-Ossowicka A, Pietrołaj M, Rumiński J (2021). A survey of neural networks usage for intrusion detection systems. Journal of Ambient Intelligence and Humanized Computing.

[ref-43] Dutta V, Choras M, Pawlicki M, Kozik R (2020). Detection of cyberattacks traces in IoT data. Journal of Universal Computer Science.

[ref-44] Ebrahimi F, Javidan R, Akbari R, Hosseini Y (2025). Intrusion detection in the internet of things using convolutional neural networks: an explainable AI approach. Cybersecurity.

[ref-45] Elmubarak M, Karrar A, Hassan N (2019). Implementation hybrid (NIDS) system using anomaly holt-winter algorithm and signature based scheme. International Journal of Advances in Scientific Research and Engineering.

[ref-46] Elrawy MF, Awad AI, Hamed HFA (2018). Intrusion detection systems for IoT-based smart environments: a survey. Journal of Cloud Computing.

[ref-47] Farah MB, Ahmed Y, Mahmoud H, Shah SA, Al-Kadri MO, Taramonli S, Bellekens X, Abozariba R, Idrissi M, Aneiba A (2024). A survey on blockchain technology in the maritime industry: challenges and future perspectives. Future Generation Computer Systems.

[ref-48] Ferrag MA, Maglaras L, Moschoyiannis S, Janicke H (2020). Deep learning for cyber security intrusion detection: approaches, datasets, and comparative study. Journal of Information Security and Applications.

[ref-49] Gill SH, Razzaq MA, Ahmad M, Almansour FM, Haq IU, Jhanjhi NZ, Alam MZ, Masud M (2022). Security and privacy aspects of cloud computing: a smart campus case study. Intelligent Automation & Soft Computing.

[ref-52] Göcs L, Johanyák ZC (2024). Identifying relevant features of CSE-CIC-IDS2018 dataset for the development of an intrusion detection system. Intelligent Data Analysis.

[ref-50] Gueriani A, Kheddar H, Mazari AC (2023). Deep reinforcement learning for intrusion detection in IoT: a survey.

[ref-51] Gyamfi E, Jurcut A (2022). Intrusion detection in internet of things systems: a review on design approaches leveraging multi-access edge computing, machine learning, and datasets. Sensors.

[ref-53] Haque S, El-Moussa F, Komninos N, Muttukrishnan R (2023). A systematic review of data-driven attack detection trends in IoT. Sensors.

[ref-54] Hdidou R, El Alami M (2024). Intrusion detection systems in internet of things: a recent state of the art. Journal of Theoretical and Applied Information Technology.

[ref-55] He Z, Liu C, Chu X, Wu W, Zheng M, Zhang D (2024). Dynamic domain-based collision avoidance system for autonomous ships: real experiments in coastal waters. Expert Systems with Applications.

[ref-56] Heorhiadi V, Reiter MK, Sekar V (2012). New opportunities for load balancing in network-wide intrusion detection systems.

[ref-57] Hijazi A, Alhafez N, Al-khayat I (2024). An adaptive distributed intrusion detection system in local network: hybrid classification methods. Journal of Intelligent Systems & Internet of Things.

[ref-58] Hindy H, Brosset D, Bayne E, Seeam A, Tachtatzis C, Atkinson R, Bellekens X (2018). A taxonomy and survey of intrusion detection system design techniques, network threats and datasets.

[ref-59] Holubenko V, Gaspar D, Leal R, Silva P (2025). Autonomous intrusion detection for IoT: a decentralized and privacy preserving approach. International Journal of Information Security.

[ref-60] Hotellier E (2024). Specification-based intrusion detection for hierarchical hybrid industrial control systems.

[ref-61] Huang D-W, Luo F, Bi J, Sun M (2022). An efficient hybrid IDS deployment architecture for multi-hop clustered wireless sensor networks. IEEE Transactions on Information Forensics and Security.

[ref-62] Jyothsna V, Prasad KM (2020). Anomaly-based intrusion. Computer and Network Security.

[ref-63] Kalnoor G, Gowrishankar S (2021). Markov decision process based model for performance analysis an intrusion detection system in IoT networks. Journal of Telecommunications and Information Technology.

[ref-64] Kermani MM, Zhang M, Raghunathan A, Jha NK (2013). Emerging frontiers in embedded security.

[ref-65] Khater BSAS (2021). A lightweight host-based intrusion detection system using n-gram and perceptron model for internet of things.

[ref-66] Khraisat A, Alazab A (2021). A critical review of intrusion detection systems in the internet of things: techniques, deployment strategy, validation strategy, attacks, public datasets and challenges. Cybersecurity.

[ref-67] Kikissagbe BR, Adda M (2024). Machine learning-based intrusion detection methods in IoT systems: a comprehensive review. Electronics.

[ref-68] Kim D, Kim J, Park S (2020). Artificial intelligence-based intrusion detection techniques: a review. Electronics.

[ref-69] Kimanzi R, Kimanga P, Cherori D, Gikunda PK (2024). Deep learning algorithms used in intrusion detection systems—a review.

[ref-70] Krishna RR, Priyadarshini A, Jha AV, Appasani B, Srinivasulu A, Bizon N (2021). State-of-the-art review on IoT threats and attacks: taxonomy, challenges and solutions. Sustainability.

[ref-71] Li J, Han D, Shi S, Xin X, Li K-C, Chang C-C (2025). An active client selection scheme based on blockchain for federated learning in shipping. IEEE Transactions on Intelligent Transportation Systems.

[ref-72] Li G, Sharma P, Pan L, Rajasegarar S, Karmakar C, Patterson N (2021). Deep learning algorithms for cyber security applications: a survey. Journal of Computer Security.

[ref-73] Luay M, Layeghy S, Hosseininoorbin S, Sarhan M, Moustafa N, Portmann M (2025). Temporal analysis of NetFlow datasets for network intrusion detection systems.

[ref-74] Makhdoom I (2020). Defense against integrity and privacy attacks in the internet of things.

[ref-75] Mallidi SKR, Ramisetty RR (2025). Optimizing intrusion detection for IoT: a systematic review of machine learning and deep learning approaches with feature selection and data balancing. Wiley Interdisciplinary Reviews: Data Mining and Knowledge Discovery.

[ref-76] Manocchio LD, Layeghy S, Lo WW, Kulatilleke GK, Sarhan M, Portmann M (2024). Flowtransformer: a transformer framework for flow-based network intrusion detection systems. Expert Systems with Applications.

[ref-77] Mbarek B, Ge M, Pitner T (2020). Enhanced network intrusion detection system protocol for internet of things.

[ref-78] Meng W, Lopez J, Xu S, Su C, Lu R (2021). IEEE access special section editorial: internet-of-things attacks and defenses: recent advances and challenges. IEEE Access.

[ref-79] Messaad MA, Jerad C, Sikora A (2021). AI approaches for IoT security analysis.

[ref-80] Mikuláš M, Kotuliak I (2024). Review of intrusion detection systems taxonomy, techniques, methods and future research directions.

[ref-81] Mills R, Marnerides AK, Broadbent M, Race N (2021). Practical intrusion detection of emerging threats. IEEE Transactions on Network and Service Management.

[ref-82] Mohamed TS, Aydin S (2022). IoT-based intrusion detection systems: a review. Smart Science.

[ref-83] Mohammed SH, Singh MSJ, Al-Jumaily A, Islam MT, Islam MS, Alenezi AM, Soliman MS (2025). Dual-hybrid intrusion detection system to detect false data injection in smart grids. PLOS ONE.

[ref-84] Monshizadeh M (2023). Machine learning techniques to detect known and novel cyber-attacks.

[ref-85] Msgna M (2022). Anatomy of attacks on IoT systems: review of attacks, impacts and countermeasures. Journal of Surveillance, Security and Safety.

[ref-86] Nagaraj T, Channarayappa RK (2024). An efficient security framework for intrusion detection and prevention in internet-of-things using machine learning. International Journal of Electrical and Computer Engineering.

[ref-87] Nilsson D (2020). Artificial intelligence for detecting IoT data intrusions.

[ref-88] Nisha NSG, Gulia P (2024). A review on machine learning based intrusion detection system for internet of things enabled environment. International Journal of Electrical and Computer Engineering (IJECE).

[ref-89] Örs FK, Levi A (2023). Data driven intrusion detection for 6LoWPAN based IoT systems. Ad Hoc Networks.

[ref-90] Perala S, Reddy M, Ravindran S, Gvk S, Bapat J (2023). Strategic placement of intrusion detection systems in IoT mesh networks through machine learning.

[ref-91] Potluri S (2024). A deep learning-driven framework for detecting anomalous data breaches in distributed cloud storage infrastructures. International Journal of Artificial Intelligence, Data Science, and Machine Learning.

[ref-92] Protogerou A, Papadopoulos S, Drosou A, Tzovaras D, Refanidis I (2021). A graph neural network method for distributed anomaly detection in IoT. Evolving Systems.

[ref-93] Rachel KM (2018). Intrusion detection system for home security by using internet of things. Journal of Web Development and Web Designing.

[ref-94] Raheema AQ (2023). Threat analysis in IOT network using evolutionary sparse convolute network intrusion detection system. International Journal of Online & Biomedical Engineering.

[ref-95] Rahman MM, Al Shakil S, Mustakim MR (2025). A survey on intrusion detection system in IoT networks. Cyber Security and Applications.

[ref-96] Rezapour A, GhasemiGol M, Takabi D (2024). A systematic mapping study on intrusion response systems. IEEE Access.

[ref-97] Rishiwal V, Agarwal U, Yadav M, Tanwar S, Garg D, Guizani M (2025). A new alliance of machine learning and quantum computing: concepts, attacks, and challenges in IoT networks. IEEE Internet of Things Journal.

[ref-98] Roihan A, Priyambodo TK, Ashari A (2019). A survey on protocols for internet of things.

[ref-99] Saba T, Rehman A, Sadad T, Kolivand H, Bahaj SA (2022). Anomaly-based intrusion detection system for IoT networks through deep learning model. Computers and Electrical Engineering.

[ref-100] Saheed YK, Abiodun AI, Misra S, Holone MK, Colomo-Palacios R (2022). A machine learning-based intrusion detection for detecting internet of things network attacks. Alexandria Engineering Journal.

[ref-101] Saleem AD, Abdulrahman AA (2024). Attacks detection in internet of things using machine learning techniques: a review. Journal of Applied Engineering and Technological Science.

[ref-102] Santos L, Gonçalves R, Rabadao C, Martins J (2023). A flow-based intrusion detection framework for internet of things networks. Cluster Computing.

[ref-103] Sarker IH (2023). Machine learning for intelligent data analysis and automation in cybersecurity: current and future prospects. Annals of Data Science.

[ref-104] Sasi T, Lashkari AH, Lu R, Xiong P, Iqbal S (2024). A comprehensive survey on IoT attacks: taxonomy, detection mechanisms and challenges. Journal of Information and Intelligence.

[ref-105] Satam P (2019). A methodology to design intrusion detection systems (IDS) for IoT/networking protocols.

[ref-106] Satımış H, Akleylek S, Tok ZY (2024). A systematic literature review on host-based intrusion detection systems. IEEE Access.

[ref-107] Serkov A, Tkachenko V, Kharchenko V, Pevnev V, Trubchaninova K (2020). A method to enhance the bandwidth and noise immunity of IIoT when exposed to natural and intentional electromagnetic interference.

[ref-108] Setiawan V, Soewito B (2025). Edge-IoTDistilBERT: fine-tuning DistilBERT for multi-class classification of network packet attacks.

[ref-109] Shah MFK, Md-Arshad M, Samad AA, Ghaleb FA (2023). Comparing FTP and SSH password brute force attack detection using k-nearest neighbour (k-NN) and decision tree in cloud computing. International Journal of Innovative Computing.

[ref-110] Sharafaldin I, Lashkari AH, Ghorbani AA (2018). Toward generating a new intrusion detection dataset and intrusion traffic characterization.

[ref-111] Shobana R, Geetha R (2025). Intrusion detection systems for modern cybersecurity: techniques, datasets, and open research challenges.

[ref-112] Siddiq A, Ghazwani YJ (2024). Hybrid optimized deep neural network based intrusion node detection and modified energy efficient centralized clustering routing protocol for wireless sensor network. IEEE Transactions on Consumer Electronics.

[ref-113] Singh R, Singh J (2012). A performance metrics scorecard based approach to intrusion detection system evaluation for wireless network. Global Journal of Computer Science and Technology—E: Network, Web & Security.

[ref-114] Smys S, Basar A, Wang H (2020). Hybrid intrusion detection system for internet of things (IoT). Journal of ISMAC.

[ref-119] Söderström A (2021). Anomaly-based intrusion detection using convolutional neural networks for IoT devices.

[ref-115] Spadaccino P, Cuomo F (2020). Intrusion detection systems for IoT: opportunities and challenges offered by edge computing and machine learning.

[ref-116] Sreelakshmi S, Babu AA, Lakshmipriya C, Gracious LAA, Nalini M, Subramanian RS (2024). Enhancing intrusion detection systems with machine learning.

[ref-117] Suganth S, Usha D (2018). A survey of intrusion detection system in IoT devices. International Journal of Advanced Research.

[ref-118] Szymanski TH (2022). The “cyber security via determinism” paradigm for a quantum safe zero trust deterministic internet of things (IoT). IEEE Access.

[ref-120] Taha A, Roy PK (2024). Improvising host-based intrusion detection systems by integrating open-source tools.

[ref-121] Taherdoost H (2023). Security and internet of things: benefits, challenges, and future perspectives. Electronics.

[ref-122] Tekin N, Acar A, Aris A, Uluagac AS, Gungor VC (2023). Energy consumption of on-device machine learning models for IoT intrusion detection. Internet of Things.

[ref-123] Thomas L, Bhat S (2021). Machine learning and deep learning techniques for IoT-based intrusion detection systems: a literature review. International Journal of Management, Technology, and Social Sciences.

[ref-124] Thulasiraman H, Kaushik V, Jain A (2024). Effective combining of feature selection techniques for machine learning-enabled intrusion detection system.

[ref-125] Togay C, Kasif A, Catal C, Tekinerdogan B (2021). A firewall policy anomaly detection framework for reliable network security. IEEE Transactions on Reliability.

[ref-126] Tripathy SS, Behera B (2025). A review of various datasets for machine learning algorithm-based intrusion detection system: advances and challenges.

[ref-127] Umair M, Cheema MA, Cheema O, Li H, Lu H (2021). Impact of COVID-19 on IoT adoption in healthcare, smart homes, smart buildings, smart cities, transportation and industrial IoT. Sensors.

[ref-128] Victor P, Lashkari AH, Lu R, Sasi T, Xiong P, Iqbal S (2023). IoT malware: an attribute-based taxonomy, detection mechanisms and challenges. Peer-to-Peer Networking and Applications.

[ref-129] Villafranca A, Thant KM, Tasic I, Cano M-D (2025). AI-enabled IoT intrusion detection: unified conceptual framework and research roadmap. Machine Learning and Knowledge Extraction.

[ref-130] Yang Z, Liu X, Li T, Wu D, Wang J, Zhao Y, Han H (2022). A systematic literature review of methods and datasets for anomaly-based network intrusion detection. Computers & Security.

[ref-131] Younus O, Majlesein B, Nacke R, Osahon INO, Pellegrino C, Babadi S, Tavakkolnia I, Helmers H, Haas H (2025). Multi-segment photonic power converters for energy harvesting and high-speed optical wireless communication.

[ref-132] Zhaikhan A, Kishk MA, ElSawy H, Alouini M-S (2020). Safeguarding the IoT from malware epidemics: a percolation theory approach. IEEE Internet of Things Journal.

[ref-133] Zhang X, Mathur A, Zhao L, Rahmat S, Niyaz Q, Javaid A, Yang X (2022). An early detection of android malware using system calls based machine learning model.

[ref-134] Zhong M, Lin M, Zhang C, Xu Z (2024). A survey on graph neural networks for intrusion detection systems: methods, trends and challenges. Computers & Security.

[ref-135] Zhukabayeva T, Benkhelifa E, Satybaldina D, Rehman AU (2024). Advancing IoT security: a review of intrusion detection systems challenges and emerging solutions.

